# Structural Regulation and Performance Enhancement of Carbon-Based Supercapacitors: Insights into Electrode Material Engineering

**DOI:** 10.3390/ma18020456

**Published:** 2025-01-20

**Authors:** Lu Guan, Dajin Li, Shanshan Ji, Xiuzhi Wei, Fanxiao Meng

**Affiliations:** 1 Department of Biological and Chemical Engineering, Jining Polytechnic, Jining 272037, China; 15854637213@163.com (D.L.); ansirain@163.com (S.J.); 15092758899@163.com (X.W.); 2 College of Chemical Engineering, China University of Petroleum (East China), Qingdao 266580, China; 3 Faculty of Humanities, Altai Li University, Barnaul 656099, Russia; 4 State Key Laboratory of Applied Organic Chemistry, College of Chemistry and Chemical Engineering, Lanzhou University, Lanzhou 730000, China

**Keywords:** carbon-based supercapacitors, pore structure optimization, heteroatom doping, intrinsic defect engineering, surface/interface modifications

## Abstract

The development of carbon-based supercapacitors is pivotal for advancing high energy and power density applications. This review provides a comprehensive analysis of structural regulation and performance enhancement strategies in carbon-based supercapacitors, focusing on electrode material engineering. Key areas explored include pore structure optimization, heteroatom doping, intrinsic defect engineering, and surface/interface modifications. These strategies significantly enhance electrochemical performance through increasing surface area, improving conductivity, facilitating charge transfer, introducing additional pseudocapacitive reactions, and optimizing the density of states at the Fermi level, among other mechanisms. After introducing these fundamental concepts, the review details various preparation methods and their effects on supercapacitor performance, highlighting the interplay between material structure and electrochemical properties. Challenges in scaling advanced fabrication techniques and ensuring the long-term stability of functionalized materials are discussed. Additionally, future research directions are proposed, emphasizing the development of cost-effective, scalable methods and interdisciplinary approaches to design next-generation supercapacitors, thereby meeting the growing demand for efficient and sustainable energy storage solutions.

## 1. Introduction

The swift depletion of fossil fuels is exacerbating the severity of the energy and environmental crisis [1,2,3,4]. The development of a diverse array of novel energy storage technologies presents a promising avenue for mitigating these challenges [5,6,7]. Supercapacitors, known for their high power density, rapid charge/discharge rates, and long cycle life, have emerged as key players in this landscape [8,9,10,11,12]. However, their relatively low energy density compared with batteries remains a significant challenge, prompting extensive research to enhance their performance [13,14,15].

To meet these challenges, significant focus has been placed on developing advanced electrode materials [16,17]. Electrode materials can be broadly categorized into carbon-based materials, metal oxides, and conductive polymers. Metal oxide electrodes, particularly transition metal oxides such as Co_3_O_4_ [18], MnO_2_ [19], and Fe_2_O_3_ [20], are widely studied due to their high capacitance and rich redox reactions. However, they often suffer from poor conductivity and cycle stability. Conductive polymers, like polyaniline (PANI) and polypyrrole (PPy), provide high specific capacitance through electrochemical polymerization, but their conductivity and long-term stability remain challenging. In contrast, carbon-based materials have proven to be particularly promising due to their exceptional electrical conductivity, high surface area, and tunable pore structures [21,22,23]. These characteristics make them ideal candidates for supercapacitor electrodes [24,25]. The versatility of carbon materials allows for various modifications and engineering strategies aimed at enhancing their electrochemical properties. These strategies are crucial for improving the key performance parameters, such as energy density, power density, and cycle stability, that are vital for the broader application of supercapacitors across different sectors, from portable electronics to large-scale energy storage systems.

**Figure 1:**
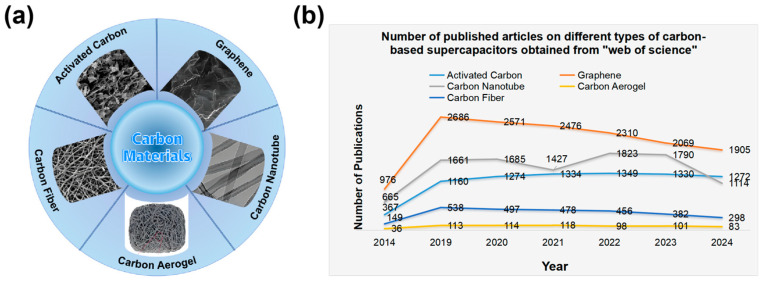
(**a**) Carbon-based materials for supercapacitors. (**b**) Trends in research publications on different carbon-based supercapacitors (2014–2024).

The evolution of supercapacitors began with a fundamental understanding of electric double-layer capacitance (EDLC), where energy storage is achieved through the electrostatic adsorption of ions on the surface of porous electrodes [26,27,28,29]. As the demand for higher performance grew, the focus expanded to include pseudocapacitors, which store energy through fast surface or near-surface redox reactions [30,31,32,33,34]. This shift necessitated a deeper exploration of material properties and the development of novel electrode materials. Significant advancements in the early 2000s, particularly in the synthesis and application of activated carbon [11,35,36,37], carbon nanotubes (CNTs) [9,38,39], graphene [40,41,42,43,44], carbon aerogels [45,46], and carbon fibers [47,48], as depicted in Figure 1a, have paved new avenues for enhancing the performance of supercapacitors. The characteristics, preparation methods, and performance advantages of these materials are meticulously summarized in Table 1. Furthermore, Figure 1b illustrates the trend in research publications on various carbon-based supercapacitors from 2014 to 2024, highlighting the significance and evolving landscape of carbon-based materials in supercapacitors. These advancements have opened up new possibilities for improving supercapacitor performance by achieving higher energy and power densities. However, these developments also underscore the need for further structural and compositional optimizations to fully harness the potential of carbon-based materials in supercapacitors.

Among the various strategies explored, adjusting the pore structure of carbon materials has been particularly effective [49,50,51]. Techniques such as chemical activation, templating, and physical activation have enabled the creation of hierarchical pore structures that combine micro-, meso-, and macropores, enhancing ion transport and storage capabilities. Additionally, heteroatom doping has shown great promise [52,53,54,55]. This involves introducing elements like nitrogen, sulfur, phosphorus, and boron into the carbon matrix to alter the electronic structure and surface chemistry of carbon materials, thereby enhancing their capacitive performance and stability. Simultaneously, the concept of intrinsic defect engineering emerged as a powerful tool for improving the electrochemical properties of carbon materials [56,57,58]. By creating and controlling defects within the carbon lattice, such as vacancies, edge defects, and topological distortions, researchers could increase the density of active sites for ion adsorption and improve ion transport mechanisms. Surface and interface modifications have also played a crucial role in the advancements of supercapacitor technology [59,60,61]. Coating carbon electrodes with conductive polymers or metal oxides not only enhances their electrochemical stability but also introduces additional pseudocapacitive reactions, thereby improving overall performance. These modifications improve the interaction between the electrode material and the electrolyte, ensuring better ion transport and higher capacitance.

This review synthesizes recent advancements in carbon-based supercapacitors and offers a comprehensive analysis of various material engineering strategies designed to enhance their performance. It highlights the innovative approaches in optimizing the structure and composition of carbon materials, focusing on key developments and emerging trends in the field. By integrating multiple techniques, this review provides valuable insights into how these strategies can overcome existing limitations and improve the energy storage capabilities of supercapacitors. Ultimately, it presents a roadmap for designing next-generation supercapacitors, emphasizing their crucial role in addressing current energy storage challenges and contributing to sustainable energy systems.

## 2. Pore Structure

The pore structure of carbon materials is a fundamental aspect that significantly influences the performance of supercapacitors [35,62]. The specific surface area and pore size distribution determine the accessibility and transport of electrolyte ions within the electrode, thereby affecting the capacitance, energy density, and power density of the supercapacitor. Pores in carbon materials are typically classified into three categories: micropores (<2 nm), mesopores (2–50 nm), and macropores (>50 nm). Each type of pore plays a distinct role in the electrochemical performance. Micropores provide a large surface area for ion adsorption, which is crucial for high capacitance. Mesopores facilitate ion transport, reducing resistance and enhancing power density, while macropores serve as reservoirs that supply ions to smaller pores, improving overall ion diffusion and accessibility [63].

Effective pore structure engineering can be accomplished through a variety of activation, templating, and other synthesis methods, which are instrumental in customizing the pore size and distribution to enhance electrochemical performance. Techniques such as physical and chemical activation, as well as template-assisted synthesis, are all capable of creating hierarchical pore structures that integrate the benefits of diverse pore dimensions. These multi-scale structures are crucial for attaining a synergistic balance between high energy storage capacity and swift charge–discharge capabilities. Consequently, a profound comprehension and precise control over the mechanisms of pore formation are imperative for the advancement of state-of-the-art carbon-based supercapacitors that exhibit exceptional performance.

### 2.1. Pore Formation Strategies

The development of porous carbon materials involves several activation methods that tailor the pore structure to enhance supercapacitor performance. These methods are crucial for achieving the desired pore size distribution and surface properties. Here, we discuss four primary activation techniques: physical activation, chemical activation, template methods, and in-situ templating.

#### 2.1.1. Chemical Activation

Chemical activation is a widely adopted method for fabricating porous carbon materials, valued for its ability to tailor the pore structure and significantly enhance specific surface areas [64,65]. Compared with physical activation, chemical activation presents numerous benefits: it achieves higher carbon yields, operates at comparatively lower temperatures, and results in porous carbon with elevated mesopore ratios. This technique involves impregnating a carbon precursor with chemical activating agents, followed by carbonization at elevated temperatures, typically between 400 °C to 800 °C. Basic chemicals, such as KOH, KHCO_3_, NaHCO_3_, K_2_CO_3_, and ZnCl_2_ as well as acids, such as H_3_PO_4_, H_2_SO_4_, and HCl, are typical chemical agents utilized in the chemical activation process of carbon [66,67,68]. The choice of chemical and its concentration play a crucial role in determining the nature and distribution of the pores developed. Understanding the specific effects of these agents on the activation process is essential for tailoring porous carbons with desired properties for various applications.

The process of chemical activation facilitates the development of a wide range of pore sizes, from micropores to mesopores, making it particularly effective for applications requiring high surface area and porosity. For instance, KOH is known for its ability to etch away carbon atoms selectively, expanding existing pores and creating new ones, thereby increasing the total pore volume and surface area available for electrolyte access in energy storage applications. For example, Wang et al. employed a strategic chemical activation using KOH to tailor the pore structure of grape-derived carbon, resulting in a honeycomb-like porous carbon with a hierarchical micro/mesoporous structure. This method effectively increased the specific surface area to 1268 m^2^ g^−1^ [69]. Charoensook et al. utilized a two-step chemical activation process involving carbonization and KOH treatment to regulate the pore structure of rice straw-derived activated carbon (AC). By varying the KOH concentration and activation temperature, they successfully tailored the pore size and enhanced the specific surface area (SSA) to 2651 m^2^ g^−1^ [70].

**Figure 2:**
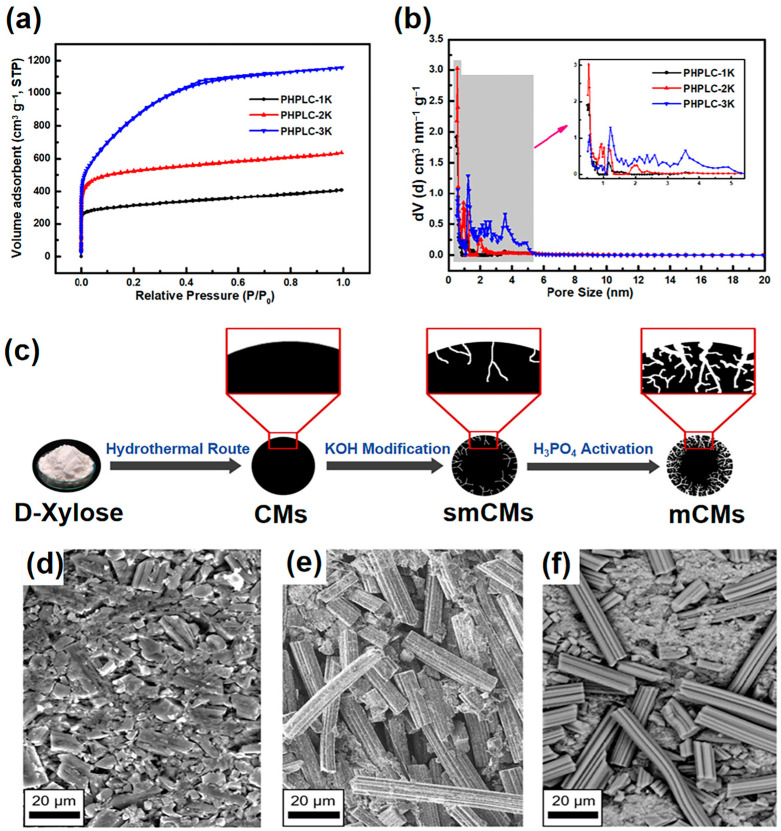
(**a**) The nitrogen adsorption–desorption curves and (**b**) the distribution of pore sizes within the phosphoric acid plus hydrogen peroxide (PHP) lignin-derived carbons (PHPLCs) [71]. (**c**) Schematic diagram of the pore formation mechanism on the carbon surface through sequential KOH-H_3_PO_4_ activation [74]. SEM micrographs of the electrode produced from the ACs activated with (**d**) CO_2_, (**e**) H_2_O and (**f**) KOH [75].

Chemical activation also allows for the control over the hierarchical structure of the pores. By adjusting the activation conditions, such as temperature, time, and the type of chemical used, researchers can optimize the pore structure for specific applications. For example, a higher concentration of KOH can lead to a larger mesopore structure, which is beneficial for faster ion transport and hence higher power densities in supercapacitors. The study by Wan et al. have demonstrated that adjusting the KOH-to-lignin ratio during chemical activation profoundly influences the pore structure of the resulting carbon materials [71]. SEM imaging confirmed the formation of a 3D porous network in all samples, which was crucial for enhancing ion diffusion in supercapacitors. Notably, a higher KOH/lignin ratio produced PHPLC-3K with an increased surface pore distribution, transitioning from a predominantly microporous structure in PHPLC-1K and PHPLC-2K to a hierarchical pore system in PHPLC-3K. This was quantified by a marked increase in specific surface area (from 1179 to 3094 m^2^ g^−1^) and total pore volume (from 0.63 to 1.72 cm^3^ g^−1^), as illustrated in Figure 2a,b. The larger SSA and hierarchical porosity of PHPLC-3K, with a wider pore size distribution (0.5 to 6 nm), were found to be instrumental in its superior electrochemical performance, making it an excellent candidate for high-performance supercapacitor electrodes. Furthermore, Taer and co-workers investigated the effect of ZnCl_2_ concentration on the porous structure of activated carbon monolith derived from the green stem of cassava (GSC). By altering the ZnCl_2_ concentration from 0.1 M to 0.7 M, they controlled the formation of micropores and mesopores. The sample activated with 0.3 M ZnCl_2_ demonstrated an optimal nanosheet and nanofiber structure, indicative of improved pore characteristics [72]. In another example, Yakaboylu et al. demonstrated that, by adjusting the chemical pretreatment time with KOH, the pore structure of activated carbons derived from Miscanthus grass could be controlled. This led to an optimal pretreatment duration of 12–18 h, resulting in enhanced micropore volume (0.26 cm^3^ g^−1^) and surface area (639 m^2^ g^−1^), as well as a rich oxygen content (18 at.%) [73].

Furthermore, the interaction between the chemical agent and the carbon matrix often introduces functional groups to the surface of the carbon. These groups can enhance the wettability of the carbon, which is beneficial for supercapacitors as it improves the interface between the electrolyte and the carbon electrodes. This modification is critical for enhancing the capacitive performance by facilitating faster and more efficient ion transport within the electrode material. Waribam et al. employed a sequential chemical activation method involving potassium hydroxide (KOH) followed by phosphoric acid (H_3_PO_4_) to prepare the porous structure of activated carbon microspheres (Figure 2c) [74]. After H_3_PO_4_ activation, phosphorous-containing functional groups, specifically C-O-PO_3_ and C-PO_3_, were identified on the surface of the carbon microspheres. These groups, originating from the reaction with H_3_PO_4_, introduced acidic sites onto the carbon surface, which played a role in enhancing the electrochemical performance by promoting charge–charge interactions and increasing the specific capacitance of the material for supercapacitor applications.

In conclusion, chemical activation stands out as a versatile and effective method for the production of porous carbons. It offers extensive control over the pore size, volume, and functionality, which can be tuned to meet the specific requirements of various applications, thereby making it a pivotal technique in the advancement of carbon-based technologies.

#### 2.1.2. Physical Activation

Physical activation is a pivotal method for engineering the pore structure of carbon materials, this process primarily involves exposing carbonaceous precursors to activating agents such as steam or carbon dioxide at elevated temperatures, typically ranging from 800 °C to 1000 °C. The choice of these gases is crucial as they partially oxidize the carbon matrix, creating pores through the selective removal of carbon atoms [76,77].

For instance, Lee et al. synthesized carbon aerogels (CAs) via a sol–gel polymerization method [78]. Subsequently, to augment porosity and introduce micropores, the carbonized CAs underwent physical activation with carbon dioxide (CO_2_) serving as the activating agent at 900 °C for different time periods (30, 45, 60, and 75 min). This CO_2_ activation process induced the formation of microporosity and the transformation of existing micropores into mesopores. Notably, the specific surface area and pore volume reached their maxima at an activation time of 60 min. The resultant activated carbon aerogels (ACAs) exhibited high specific surface areas (2503 m^2^ g^−1^) and total pore volumes (1.604 cm^3^ g^−1^), which are pivotal factors for attaining high electrical double-layer capacitance.

Beyond single gas activation, carbon materials with more intricate pore architectures can be fabricated through co-activation methods. Zou’s research team systematically explored the influence of CO_2_ and H_2_O co-activation on the specific capacitance, morphology, pore structure, and physicochemical properties of the activated carbons, as well as their subsequent supercapacitor performance [79]. A hierarchical porous structure, rich in both micropores and mesopores, along with a well-developed graphite microcrystalline structure, was formed in the carbon co-activated by CO_2_ and H_2_O at 800 °C. This co-activation strategy not only led to a high char yield but also generated oxygen- and nitrogen-containing functional groups. These functional groups are advantageous for enhancing the wettability of the electrode and facilitating ion diffusion within the pores. Significantly, the activated carbon prepared via this co-activation method exhibited superior electrochemical performance compared with those activated by CO_2_ or H_2_O alone, as manifested by an ideal coulombic efficiency and specific capacitance.

The control over the physical activation process allows for a tailored pore structure, crucial for optimizing the material’s properties. Key parameters, such as activation temperature, gas concentration, residence time, and gas flow rate, are meticulously adjusted to regulate the extent of burn-off and the development of pore size and distribution. Ding et al. achieved the regulation of pore structure in carbon materials through a physical activation process utilizing carbon dioxide (CO_2_). By adjusting the concentration of CO_2_ during the activation stage, they controlled the specific surface area, pore size distribution, and the formation of nitrogen-containing functional groups in the biochar derived from bean pulp. The one-step carbonization and CO_2_ activation at 1073 K resulted in a significant enhancement of the biochar’s specific surface area, which reached a maximum of 558.2 m^2^ g^−^^1^ for the sample activated under 100 vol% CO_2_. Additionally, the nitrogen content increased from 5.0% to 10.0% as the CO_2_ concentration increased from 0 to 50 vol%, effectively introducing various nitrogenous functional groups that contributed to the pseudocapacitance of the material. This method demonstrated a sustainable and efficient way to produce activated carbon with tailored porosity and high nitrogen doping for supercapacitor applications [51].

A well-controlled physical activation process can significantly enhance the accessibility of the internal surface area of carbon materials. This is particularly beneficial for supercapacitors, where the speed of ion transport to and from the surface governs the device’s charging and discharging rates. For instance, an optimized porous network facilitates the swift movement of electrolyte ions, ensuring more efficient energy storage and faster power delivery. Furthermore, the ability to control the pore size distribution between micro-, meso-, and sometimes macropores enables the tailoring of carbon materials to specific needs, enhancing performance in targeted applications.

Furthermore, physical activation is a milder process than chemical activation. Breitenbach et al. compared the activation of viscose-based carbon fibers using KOH, CO_2_, and H_2_O [75]. They found that all methods produced high surface area fibers, but CO_2_ and H_2_O preserved the fiber structure (Figure 2d–f), leading to lower resistance supercapacitors. In contrast, KOH activation resulted in a powdery form with higher resistance. The combined CO_2_ and H_2_O activation offered high purity activated carbon fibers (ACFs) with superior electrochemical performance, achieving an energy density of 42 W h kg^−1^. Additionally, the environmental aspect of physical activation is noteworthy. Unlike chemical activation, which often involves corrosive chemicals that can lead to hazardous waste issues, physical activation uses gases that can be handled and disposed of more safely and sustainably. This aspect makes physical activation a more environmentally friendly option for producing advanced porous carbons.

In summary, physical activation offers a robust and versatile approach to modifying the pore architecture of carbon materials. By fine-tuning the process parameters, manufacturers can produce carbons with specific porosities that are ideal for their intended applications, combining efficiency with environmental sustainability.

#### 2.1.3. Template Method

The template method, a sophisticated approach for engineering porous carbon materials, provides precise control over the pore structure, crucial for optimizing the performance of carbon-based applications. This method involves using a template, which could be either hard or soft, to dictate the shape, size, and distribution of pores within the carbon matrix [80,81,82].

**Figure 3:**
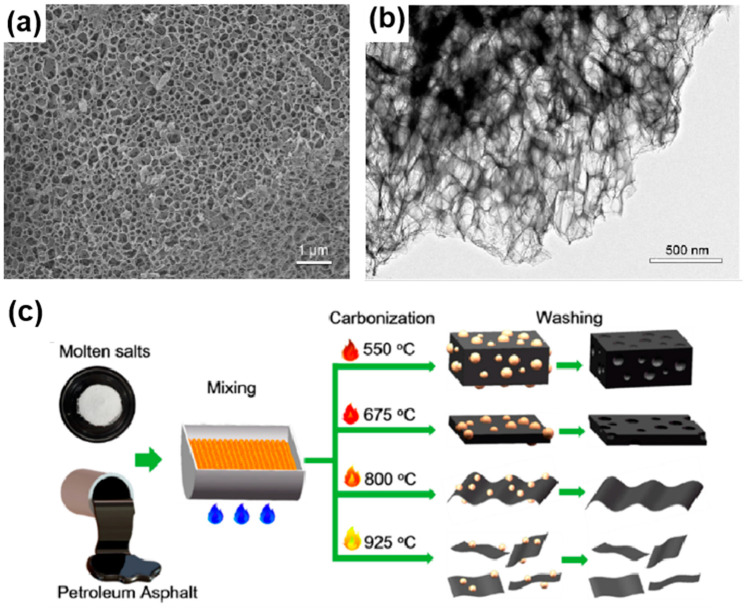
(**a**) SEM image and (**b**) TEM image of NHPC-700 [86]. (**c**) Schematic diagram of the evolution process of molten-salt carbons [87].

The hard template method is an efficient strategy for creating porous carbons with tailored pore structures and sizes. Templates like MgO, CaCO_3_, ZnO, Fe_2_O_3_, silica, and KCl are often used to produce uniform porous carbon architectures [83,84]. The carbon’s structure is largely dependent on the template’s properties. The process involves synthesizing a suitable hard template, mixing it with carbon sources, pyrolyzing at high temperatures under a specific atmosphere, and removing the template with an acid or base wash. For example, Jiang and colleagues developed an innovative approach to fabricate porous carbon materials by employing a ZnO template method [85]. They meticulously coated coal tar pitch onto Zn_5_(OH)_6_(CO_3_)_2_ microspheres synthesized via a hydrothermal process using zinc nitrate hexahydrate and urea. Subsequently, the coated microspheres underwent carbonization and KOH activation. Through this elaborate procedure, they successfully synthesized porous carbon-sheet microspheres with a hierarchical pore structure and a large specific surface area, exhibiting a specific capacitance as high as 313 F g^−^^1^ at 1 A g^−^^1^, which clearly demonstrates enhanced electrochemical performance and great potential for supercapacitor applications. In another example, Mo et al. developed a method utilizing magnesium oxide (MgO) as a template to fabricate heteroatom-doped hierarchical porous carbon (NHPC) [86]. The process began with the synthesis of an MgO template through sonication and calcination. Subsequently, anionic polyacrylamide was combined with the MgO template via electrostatic self-assembly, followed by freeze-drying and carbonization at various temperatures under a nitrogen atmosphere. The final step involved acid and water washing to remove the MgO, yielding the NHPC. This approach resulted in a 3D interconnected hierarchical porous structure with a high specific surface area and abundant heteroatom doping (Figure 3a,b), which significantly enhanced the electrochemical performance for supercapacitor applications.

In soft template synthesis, organic molecules with functional groups serve as templates, enabling interactions like hydrogen bonding and electrostatic forces in a solvent. With the addition of a suitable solvent, these soft templates form micelles that coat carbon precursor molecules. Upon carbonization, micelles decompose, leaving behind porous carbon structures. Pore characteristics are adjustable via the solvent-to-micelle ratio. Effective soft templates should self-assemble into nanostructures, contain components that form micro- or mesopores upon carbonization, and remain stable until the carbon precursor solidifies to ensure the formation of the desired pore architecture. Xue et al. developed a soft-template approach utilizing deep-eutectic solvents (DESs) to fabricate N/O self-doped hollow carbon nanorods (HCNs). The process commenced with the condensation of DES, which simultaneously acted as a carbon source, self-template, and self-dopant. Upon carbonization, the DES-derived HCNs exhibited a hollow structure with micro/mesoporous walls. The soft-template method allowed precise tuning of the porosity and heteroatom doping, which significantly influenced the electrochemical performance, yielding a high energy density supercapacitor with excellent cycling stability [88].

Both hard and soft templating approaches allow for the synthesis of carbons with tailored pore structures. These structures significantly enhance the performance of energy storage devices by providing improved pathways for ion transport and increased surface areas for electrochemical reactions. Moreover, the uniform and controlled pore structures achieved through templating contribute to the repeatability and scalability of the carbon production process, crucial for commercial applications.

The selection of the templating agent and the conditions under which the carbonization and template removal processes occur are critical for achieving the desired pore architecture. Thus, the template method not only underscores the importance of material design in advanced applications but also highlights the intricate balance required between material structure and functionality.

#### 2.1.4. In-Situ Templating

As for the in-situ templating synthesis, the formation of templates typically occurs via decomposition, melting, and polymerization processes at elevated temperatures. Concurrently, the precursors for porous carbons are melted and engage with these in-situ generated templates, resulting in the creation of carbon materials that exhibit a variety of distinct pore structures. Additionally, the formation of in-situ templates is primarily categorized into three types: top-down, state-change, and bottom-up approaches [89].

Top-down templating involves the utilization of compounds that, upon heating, decompose or transform into thermally stable entities with defined shapes and structures. These entities serve as frameworks for the introduction of carbon sources, which subsequently undergo thermal polymerization to form carbon materials. In certain instances, these templates can also act as catalysts for the polymerization process, thereby controlling the crystallinity of the resulting carbon. Commonly employed for this purpose are chemical compounds that are prone to thermal decomposition, such as citrates, oxalates, basic carbonates, and acetates. For instance, Yang and colleagues have demonstrated the creation of a 3D carbon framework with substantial interlayer distances and enhanced conductivity by calcining sodium citrate [90]. State-change templates refer to salts that, upon melting at elevated temperatures, can significantly influence the pyrolysis of organic precursors, thereby crafting porous carbon materials with targeted architectures. Utilizing this approach, Wu’s group have generated a suite of porous carbon substances. They employed molten-salt techniques, leveraging petroleum asphalt—a cost-effective byproduct from oil refineries—as the carbon source. Specifically, they selected the eutectic mixture of KCl and CaCl_2_, which melts at 600 °C, to establish a medium conducive to the formation of ultra-thin carbon nanosheets [87]. Using this technique, they have been able, not only of fabricating porous carbon particles with a high porosity and a large specific surface area, but also to modulate the structure of these materials, ranging from porous carbon particles to thick carbon nanoplates and ultrathin carbon nanosheets, by incrementally elevating the heating temperature, as illustrated in Figure 3c. The bottom-up in-situ templating involves the transformation of small molecular precursors into nano- or micro-scale structures. Notably, graphitic carbon nitride (g-C_3_N_4_) stands out as a quintessential example of such structures, typically synthesized via thermal polycondensation of precursors like melamine, dicyandiamide, and urea [91,92].

The benefits of in-situ templating are manifold. It allows for the simultaneous achievement of carbonization and pore formation, significantly simplifying the manufacturing process and enhancing the efficiency of scale-up operations. Additionally, one of the key advantages of in-situ templating is the ability to produce carbons with hierarchical pore structures. These structures combine micro, meso, and macropores, which are essential for applications requiring rapid mass transport and high surface accessibility. Furthermore, in-situ templating facilitates the incorporation of functional groups or doping elements into the carbon matrix. As the precursor decomposes, it can also integrate elements like nitrogen, sulfur, or phosphorus, enhancing the electrochemical properties of the carbon material. This method thus not only controls the physical attributes of the pores but also tailors the chemical surface properties, optimizing the interaction between the electrode material and the electrolyte.

In conclusion, in-situ templating represents a highly efficient and versatile approach to the synthesis of advanced porous carbons. By allowing for precise control over both the microstructure and chemical properties of the carbon, this technique meets the critical demands of high-performance applications, making it a pivotal development in the field of material science.

Having delved into the foundational aspects of each pore formation strategy, it is imperative to present a comparative analysis to achieve a thorough understanding. Table 2, detailed subsequently, offers a methodical juxtaposition of physical activation, chemical activation, template-based strategies, and in-situ methods. This comparison encompasses a broad spectrum of considerations including the underlying activation principles, the precision of pore structure control, the consequential effects on supercapacitor performance, and an assessment of the respective advantages and disadvantages inherent to each approach.

### 2.2. Pore Characteristics and Supercapacitor Performance

The manipulation of pore structure in carbon materials is essential for the enhanced performance of supercapacitors, as it directly affects the capacitance, energy density, power density, and cycle stability [93,94,95].

**Figure 4:**
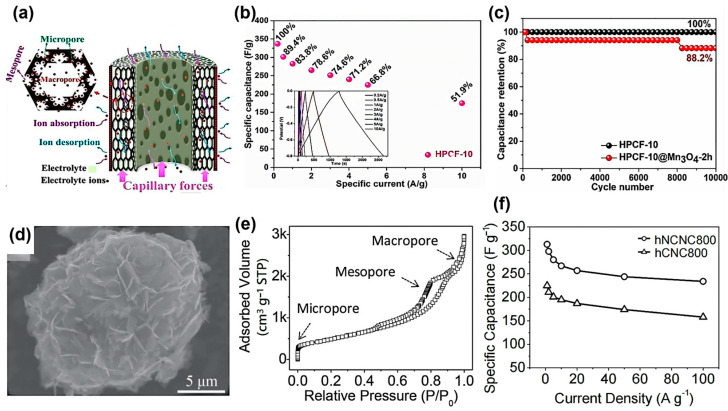
(**a**) EDLC mechanism of self-standing HPCF-10 electrode. (**b**) Rate performance curves of HPCF-10 electrode from 0.2 A g^−1^ to 10 A g^−1^, (**c**) cycling stability of HPCF-10 at the current density of 10 A g^−1^ [96]. (**d**) SEM image, (**e**) nitrogen adsorption and desorption isotherm and (**f**) gravimetric capacitances at different charge–discharge current densities of hNCNC800 [97].

Liu et al. conducted a detailed study on the fabrication of hollow porous carbon fibers (HPCFs) with a tunable hierarchical structure [96]. The authors meticulously engineered the hierarchical structure, which includes macro-, meso-, and micro-pores, through a wet-spinning process combined with chemical foaming, followed by carbonization and activation, as illustrated in Figure 4a. This approach resulted in a high specific surface area and substantial total pore volume, both of which are crucial for enhancing supercapacitor capacitance.

The micropores in the HPCF are critical for ion adsorption due to their large surface area, which significantly boosts capacitance and energy density by enabling more efficient electrostatic ion storage. The rate capability and power density are also augmented by the presence of mesopores and macropores, which expedite ion transport and reduce internal resistance, facilitating faster charging and discharging cycles. A hierarchical pore structure that integrates these different sizes of pores is beneficial for cycle stability, providing efficient ion pathways while minimizing structural changes over time, thus extending the supercapacitor’s operational life. Moreover, a uniform and interconnected pore network ensures comprehensive electrolyte penetration throughout the electrode, maximizing the area for electrochemical reactions and mitigating “dead spots”. Larger pore volumes and well-distributed pores also help in maintaining the physical and electrochemical stability of the electrodes by accommodating the volume changes during ion adsorption and desorption processes. The self-standing HPCF10 electrode exhibited outstanding performance, providing a significant specific capacitance of 283 F g^−1^ at a current density of 1 A g^−1^ (Figure 4b). It also demonstrated superior long-term cyclability, retaining 100% of its initial capacitance after 10,000 charge–discharge cycles (Figure 4c). Supercapacitors crafted from HPCF thus possess considerable potential for enhancing energy storage technology. Furthermore, Zhao et al. have meticulously engineered hierarchical nitrogen-doped carbon nanocages (hNCNCs) [97], as shown in Figure 4d, revealing the significant impact of tailored pore structures on the electrochemical performance of supercapacitors. This multiscale porosity not only ensures a high specific surface area (Figure 4e) for ion adsorption but also facilitates ion transport and diffusion by serving as ion reservoirs and conduits. The interconnected pore network of hNCNCs, as synthesized at optimal temperatures, provided a substantial specific capacitance of up to 313 F g^−1^ at 1 A g^−1^ (Figure 4f), reflecting the synergistic impact of well-tailored pore architecture on charge storage and power delivery.

In essence, the precise engineering of pore structure in carbon materials is indispensable for the optimization of supercapacitor performance across various metrics. Utilizing sophisticated material engineering techniques, it is possible to exert fine control over porosity at all levels—micro, meso, and macro—allowing for the customization of supercapacitors to fulfill the specific demands of different applications. This meticulous design approach is pivotal in achieving supercapacitors with superior energy storage and power delivery capabilities.

## 3. Heteroatom Doping

Beyond the optimization of pore structures, heteroatom doping represents a pivotal approach for significantly enhancing the performance of carbon-based supercapacitors. Heteroatom doping involves the intentional introduction of foreign atoms into the carbon matrix, which significantly enhances the electrochemical properties of carbon-based materials [98]. By incorporating elements such as nitrogen [99,100], sulfur [101,102], phosphorus [103,104,105], and boron [106,107], the electronic structure, conductivity, and surface chemistry of the carbon material can be tailored to improve performance in supercapacitor applications. This approach not only enhances the intrinsic properties of the carbon materials but also introduces additional pseudocapacitive effects, leading to higher capacitance and energy density. Over the past few decades, research has demonstrated that heteroatom doping can effectively modulate the electron distribution within the carbon framework, thereby facilitating faster ion transport and improving overall electrochemical stability. The diverse effects of different heteroatoms and their synergies have opened up new avenues for optimizing supercapacitor electrodes.

### 3.1. Doping Strategies

Doping, which involves the intentional introduction of foreign atoms or molecules into a material, is a widely utilized strategy to precisely tailor the properties of carbon-based materials for a range of applications. In the context of supercapacitors, doping is instrumental in modulating the electrochemical performance of carbon electrodes. This section provides a comprehensive overview of various doping strategies employed in the synthesis of carbon-based materials specifically for supercapacitor applications, highlighting their impact on enhancing the overall efficiency and performance of the devices.

#### 3.1.1. Post-Treatment Doping

Post-treatment doping refers to the method of modifying the surface of porous carbon materials with heteroatom-rich small molecules or polymers (hereafter referred to as dopants) to achieve heteroatom doping in originally undoped carbon materials. Commonly used small molecule dopants for post-treatment include nitrogen-containing molecules such as ammonia, urea, and cyanamide; phosphorus-containing molecules like phosphoric acid, phosphates, and phytic acid; sulfur-containing molecules such as elemental sulfur, hydrogen sulfide, thiourea, and persulfates; and boron-containing molecules like boric acid and borates. For polymers, nitrogen-containing polymers such as polyaniline, polypyrrole, and melamine; sulfur-containing polymers like polythiophene and sulfone polymers; and phosphorus-containing polymers such as polyphosphazene are typically used.

**Figure 5:**
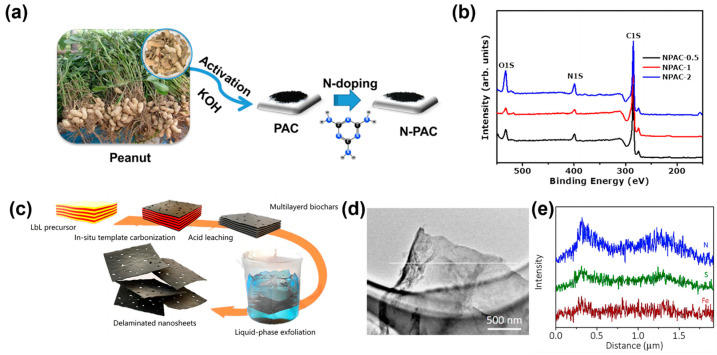
(**a**) Schematic illustration of the synthesis process for the N-doping of the activated carbon and (**b**) XPS survey spectra of the N doped samples [111]. (**c**) Synthetic process of delaminated carbon nanosheets, (**d**) EDX-TEM image of Fe-N-S-CNS and (**e**) corresponding line profile concentrations for N, S, and Fe [112].

Based on the state of the dopants during the doping process, post-treatment doping can be categorized into two main types:

Dry post-treatment: This method involves mixing the porous carbon materials with solid dopants followed by high-temperature pyrolysis or directly treating the porous carbon materials in an atmosphere of gaseous dopants [108,109,110]. This technique is characterized by its simplicity and broad applicability and remains a common strategy for preparing heteroatom-doped porous carbon materials. During pyrolysis, the dopant decomposes to release a large volume of heteroatom-containing gases, serving a dual purpose of doping and mildly activating the carbon material. Optimization of the carbon-to-dopant ratio, along with pyrolysis temperature and duration, allows effective control over the heteroatom content, pore structure, and specific surface area of the doped carbon materials. For example, Sylla et al. detailed the synthesis of ex-situ nitrogen-doped porous carbons (NPAC) using biomass waste, specifically peanut shells, with melamine as the nitrogen source [111]. As illustrated in Figure 5a, this two-step process began with the chemical activation of the peanut shells using KOH, followed by nitrogen doping of the activated carbon. The results were found to illustrate that the optimal amount of KOH relative to peanut shells yielded a material with superior capacitance performance, achieving a specific surface area of 1442 m^2^ g^−1^ and a nitrogen content of 3.2 at.% (Figure 5b). The treated carbons exhibited impressive electrochemical properties, notably a specific capacitance per electrode of 251.2 F g^−1^ at 1 A g^−1^ in aqueous electrolyte and maintained significant stability over 20,000 charge–discharge cycles. These findings underscore the potential of ex-situ doping to enhance both the structural and functional properties of biomass-derived carbons for energy storage applications.

Wet post-treatment: This method involves immersing the porous carbon materials in a solution or dispersion of the dopants to ensure thorough modification, followed by separation, drying, or further pyrolysis to prepare the doped porous carbon. Compared with the dry post-treatment, the wet method uses liquid-phase mixing, which not only enhances the mixing efficiency between the carbon material and the dopant for thorough modification but also facilitates large-scale industrial production. This doping method has gained considerable attention in recent years and is extensively used in the preparation of doped graphene, carbon nanotubes, and carbon fibers. In exploring wet post-treatment strategies, the study by Tian et al. exemplifies the liquid-phase exfoliation of layered biochars into multifunctional heteroatom co-doped graphene-like carbon nanosheets (Figure 5c) [112]. This innovative approach employs a liquid medium to delaminate and dope the carbon material, leading to enhanced textural and electrochemical properties. The resulting carbon nanosheets (Fe-N-S-CNS), co-doped with Fe, N, and S (Figure 5d,e), exhibit a high specific surface area and exceptional electrochemical performance, serving as efficient electrodes for oxygen reduction and supercapacitive energy storage. This method not only underscores the versatility of wet post-treatment techniques but also highlights the potential for scaling up the production of high-performance carbon-based materials for advanced applications.

Both methods have been pivotal in advancing the functionalities of doped carbon materials, contributing significantly to their application in supercapacitors and other high-performance devices.

#### 3.1.2. In-Situ Doping

In-situ doping involves incorporating heteroatoms directly into carbon materials during the synthesis process, using biomass, organic molecules, or polymers rich in heteroatoms as precursors [113]. This method embeds heteroatoms uniformly into the carbon framework, achieving high concentrations crucial for the electrochemical performance of the materials. Compared with post-treatment doping, in-situ doping methods are efficient and ideal for fabricating high-performance heteroatom-doped porous carbon materials.

**Figure 6:**
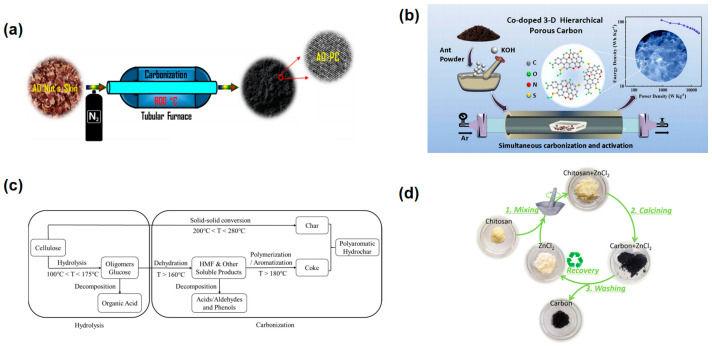
Schematic illustration of the synthesis process for (**a**) the nut skin waste-derived porous carbon [114] and the (**b**) 3D hierarchical porous carbons [116]. (**c**) The reaction path for CL carbonization under hydrothermal conditions [117]. (**d**) The synthetic process of nitrogen-doped porous carbon materials prepared by salt template method [118].

Based on the method of integrating the dopants during the synthesis process, in-situ doping can be categorized into several main types, as follows:

Direct pyrolysis: Direct pyrolysis involves thermally decomposing heteroatom-rich precursors at temperatures ranging from 500 °C to 1000 °C to fabricate doped porous carbon materials. This method is straightforward and widely used for substances like biomass, small organic molecules, and polymers, producing high-quality doped carbons directly from the carbonization of these precursors. As an example, Atchudan et al. developed nitrogen-, oxygen-, and sulfur-doped porous carbons from cashew nut skin waste through direct pyrolysis [114]. The resulting carbon materials showcased interconnected micropore/mesopore structures, as shown in Figure 6a, with a specific surface area of 615 m^2^ g^−1^ and a high specific capacitance of 193 F g^−1^ at 0.5 A g^−1^. These carbons also demonstrated excellent cyclic stability, retaining 97% of their capacitance after 10,000 cycles. In another study, imidazolinium polymers were carbonized to produce N/O-doped carbon materials that exhibited a high specific capacitance of 199.1 F g^−1^ at 1 A g^−1^ and an energy density of 27.7 Wh kg^−1^ at a power density of 496.5 W kg^−1^. The materials displayed superior cycling stability and pseudocapacitive performance, attributed to the diverse pseudocapacitive active NO species [115].

Activation methods: Activation methods for in-situ doping involve using heteroatom-rich substances as precursors, which are then activated through thermal processes. This can be undertaken in a single-step process where carbonization and activation occur simultaneously, or a two-step process involving carbonization followed by activation. The activation can be physical—using agents like CO_2_, steam, or air to create pores at high temperatures—or chemical—using agents like KOH, NaOH, or ZnCl_2_ to chemically react and etch pores during the pyrolysis process. This method allows precise control over the heteroatom content, surface area, and pore distribution, although it might result in lower carbon yields and potential loss of heteroatoms. To give an example, Zhao and colleagues developed a one-step KOH activation method to fabricate O-N-S tri-doped hierarchical porous carbons (HPCs) from ant powder (Figure 6b) [116]. These HPCs possess a 3D interconnected porous structure, an ultra-high specific surface area of 2650 m^2^ g^−^^1^, and significant heteroatom doping, which collectively contribute to their superior supercapacitor performance, achieving a specific capacitance of 576 F g^−^^1^ and an energy density of 107 Wh kg^−^^1^, setting a new standard for biomass-derived carbon supercapacitors.

Hydrothermal method: The hydrothermal method involves treating heteroatom-rich precursors under high pressure in autoclaves followed by high-temperature pyrolysis to produce doped porous carbons. This technique is particularly beneficial for biomass-based materials and typically includes the addition of activators or dopants in the solvent, enhancing the doping effect and leading to materials with high heteroatom content and developed pore structures. For instance, Liu et al. explored the use of hydrothermal carbonization (HTC) for enhancing supercapacitor carbon production by utilizing cellulose doped with ammonium sulfate and thiourea (Figure 6c) [117]. Conducted at 240 °C for one hour, the HTC process enabled the effective integration of heteroatoms such as nitrogen into the carbon structure, significantly improving its electrochemical properties. After KOH activation, the treated carbon exhibited a remarkable specific surface area of 952.27 m^2^ g^−1^ and achieved a specific capacitance of 235.8 F g^−1^ at a current density of 1 A g^−1^. Impressively, after 20,000 charge–discharge cycles at a current density of 10 A g^−1^, the material maintained a capacitance retention rate of nearly 100%, demonstrating its potential for high-performance energy storage applications.

Salt templating: Salt templating involves mixing heteroatom-rich precursors with high-melting-point inorganic salts and then carbonizing the mixture in an air or inert atmosphere. The precursor is encapsulated within the molten salt, decomposing gradually; the salt acts both as a physical barrier enhancing carbon yield and a template that influences the morphology of the resulting carbon. Commonly used salts include NaCl, KCl, CaCl_2_, and BaCl_2_. This method is simple, green, and effective, allowing control over the carbon’s morphology and composition by varying the type and amount of salt and processing conditions. As an example of this, Deng et al. demonstrated the synthesis of nitrogen-doped carbon materials using a molten-salt method, which showcases a promising approach for supercapacitor applications [118]. Utilizing chitosan as a nitrogen-containing precursor and ZnCl_2_ as a molten salt, they achieved the synthesis of hierarchically porous carbon at 600 °C, as depicted in Figure 6d. The resulting material possessed a specific surface area of 1582 m^2^ g^−1^ and exhibited a high nitrogen content, which contributed to a specific capacitance of 252 F g^−1^ at 0.5 A g^−1^. This performance was maintained with nearly 100% capacitance retention after 20,000 cycles at 10 A g^−1^, highlighting the material’s robust stability and potential for energy storage applications.

Besides the previously discussed methods, other notable doping strategies for enhancing supercapacitor performance include gas-phase doping, plasma treatment doping, and templating. Gas-phase doping involves introducing heteroatoms in a gaseous state during production, ensuring uniform distribution. Plasma treatment uses a plasma field to break molecular bonds and incorporate heteroatoms, enhancing surface properties and electrochemical performance. Lastly, the templating method utilizes hard or soft templates to precisely control the morphology and pore structure of carbon materials during synthesis, essential for tailored applications.

In summary, doping strategies represent versatile approaches for tailoring the properties of carbon-based materials for supercapacitor applications. By judiciously selecting doping methods and dopant species, researchers can optimize the electrochemical performance of carbon electrodes and pave the way for the development of high-performance supercapacitors for energy storage applications.

### 3.2. Electrochemical Effects of Heteroatom Doping

Incorporating heteroatoms into carbon materials significantly enhances the electrochemical performance of carbon-based electrodes. Primarily, integrating heterogeneous atoms into the carbon matrix generates positive partial charges on adjacent carbon atoms or heteroatoms with lower electronegativity, resulting in a polarized electrode surface. This polarization increases the interaction with electrolyte ions, facilitating their adsorption. Moreover, the substitution of electron-donor heteroatoms adds lone pair electrons to the delocalized carbon network, boosting inherent conductivity and lowering the valence band, which supports sustained operation and high electron density at the Fermi level. Additionally, embedding pseudo-active heteroatoms improves proton adsorption and grants enhanced faradaic pseudocapacitances through electrochemical redox reactions. Furthermore, co-doping or multi-doping with various heteroatoms often results in synergistic effects from spin and charge redistribution, thereby enhancing the electroactive density and overall performance of the electrode. However, excessive or highly active heteroatom doping through various fabrication methods and conditions may result in underdeveloped porosity or structural collapse in the functionalized carbons, leading to deteriorated performance in capacitors. Consequently, substantial research efforts are directed towards optimizing the synergy between heteroatom doping and porous structure using appropriate fabrication techniques and conditions.

**Figure 7:**
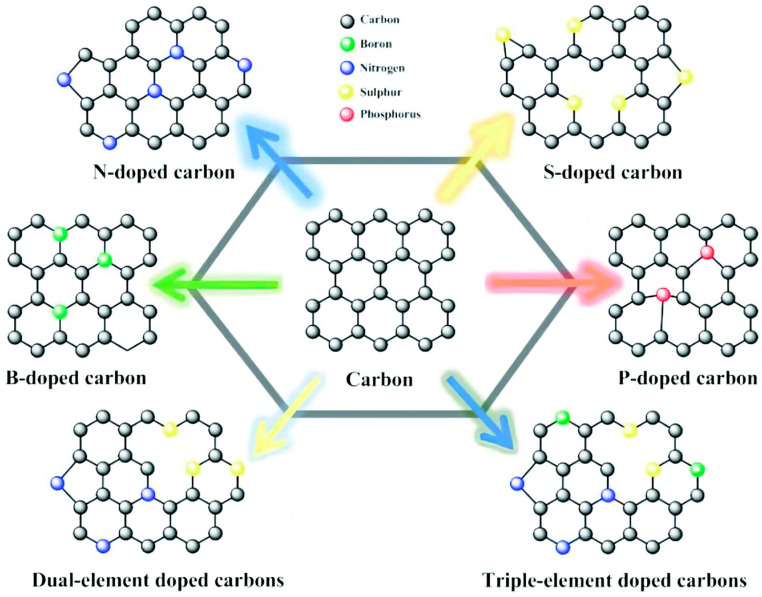
Schematic diagram of the structures of various heteroatom-doped carbons [119].

Current research on heteroatom doping primarily focuses on elements such as N, O, B, P, and S [119] (Figure 7). In the periodic table, nitrogen (N) is adjacent to carbon (C), with atomic radii that are sufficiently similar, facilitating the incorporation of nitrogen into the carbon lattice while preserving the integrity of the carbon material’s crystalline structure to the greatest extent possible. Additionally, the lone pair of electrons associated with nitrogen can increase the density of states at the Fermi level of carbon materials, resulting in n-type doping [120]. Density functional theory calculations and molecular dynamics simulations have confirmed that the introduction of additional electrons enhances the quantum capacitance of graphene near the Fermi level [121]. Consequently, nitrogen doping is currently one of the most common methods for the modification of carbon materials [122]. Oxygen doping, with its slightly larger atomic radius, induces structural defects that enhance pore distribution and surface activity. These exist predominantly as C-O-C and -COOH bonds, which improve electrochemical performance and surface wettability [123]. Boron doping, with a smaller atomic radius, maintains the sp^2^ hybridization of carbon, accelerating charge transfer and enhancing the material’s performance in aqueous electrolytes [124]. Phosphorus doping, characterized by a larger atomic radius, creates substantial structural defects, enriching the material with a high specific surface area and a diverse pore structure and, through the formation of C-P-O groups, enhances electrocatalytic activity, broadens the voltage window, and optimizes capacitive performance [125]. Lastly, sulfur doping, with its significantly larger atomic radius compared with carbon, introduces thiophene sulfur at the edge regions or defect sites of the carbon plane, creating redox reaction pseudocapacitive active sites that improve surface chemical properties [126]. Collectively, these doping elements tailor the carbon materials’ properties for applications in energy fields.

For instance, Chen et al. have demonstrated that doping carbon materials with heteroatoms such as nitrogen and oxygen leads to a substantial increase in specific capacitance, achieving up to 199.1 F g^−1^, and an energy density of 27.7 Wh kg^−1^ at a power density of 496.5 W kg^−1^. These improvements stem from the increased pseudocapacitance provided by faradaic reactions of the doped heteroatoms, alongside the enhancements in surface area and conductivity that are crucial for energy storage applications [115]. Zhao and colleagues utilized a self-doping strategy with nitrogen (N), phosphorus (P), and sulfur (S) to produce hierarchically porous carbon for supercapacitors. This method significantly increased the specific capacitance to an ultrahigh value of 525 F g^−1^ at 1 A g^−1^ and improved the energy density to 24.9 Wh kg^−1^ at 400 W kg^−1^, demonstrating substantial enhancements in electrochemical performance [127]. Manickam Minakshi’s team has made significant contributions regarding heteroatom doping in carbon materials for supercapacitors. In one study, they activated wheat straw with acid or base [128]. The acid activation introduced heteroatoms such as C-SO_x_-C and S=C into the carbon framework of wheat straw (WS) acid. This led to a specific capacitance of 162 F g^−1^ at 2 mA cm^−2^, outperforming the base-activated WS base with 106 F g^−^^1^. The heteroatoms enhanced the interaction with electrolyte ions and affected the electronic structure, thus improving the electrochemical performance. In another study, they prepared phosphorous and oxygen co-doped honeydew peel-derived activated carbon (HDP-AC) via H_3_PO_4_ activation [66]. The presence of the P and O heteroatoms in the HDP-AC, played a crucial role. It achieved a high specific capacitance (e.g., 612 F g^−1^ at 5 mV s^−1^) and maintained good performance at different scan rates and current densities. The heteroatoms also contributed to a high energy density of 66.5 Wh kg^−1^, a power density of 662 W kg^−1^, and excellent capacitance retention (98.5% after 1000 cycles) by enhancing defects, active sites, and surface wettability. To furnish a thorough comprehension of the electrochemical effects precipitated by heteroatom doping, Table 3 is presented. Table 3 offers a meticulous synthesis of how various heteroatoms influence the performance metrics of supercapacitors.

In summary, heteroatom doping provides an efficient method for improving the performance of supercapacitors. By selecting appropriate doping elements and ratios, it is possible to maximize performance while ensuring stability, thus allowing supercapacitors to exhibit greater potential in various applications, especially in scenarios requiring high energy and power densities. Future research should explore new doping elements and optimize existing doping strategies to achieve better electrochemical performance.

## 4. Intrinsic Carbon Defects

While pore structure optimization enhances ion transport and surface area, and heteroatom doping modifies electronic properties and introduces pseudocapacitance, intrinsic carbon defects offer a different pathway for performance enhancement [145,146,147]. Intrinsic carbon defects, such as vacancies, edge defects, and topological distortions, play a vital role in enhancing the electrochemical properties of carbon-based materials [148,149,150]. These defects introduce irregularities within the carbon lattice, creating additional active sites for ion adsorption and facilitating faster ion transport [151,152,153]. The presence of defects can significantly increase the surface area and reactivity of the carbon material, leading to improved capacitance and energy density in supercapacitors. By carefully engineering these defects, it is possible to optimize the electronic structure and surface chemistry of carbon materials, thereby achieving superior performance in energy storage applications. Understanding and controlling the formation of intrinsic carbon defects is crucial for the development of advanced carbon-based supercapacitors.

### 4.1. Formation Strategies

Introducing intrinsic defects into carbon materials is a critical process for enhancing their electrochemical performance. The primary methods for achieving this include plasma treatment, Nitrogen removal strategy, and ball milling. Each of these techniques offers distinct advantages and allows for precise control over the type and density of defects introduced into the carbon structure.

#### 4.1.1. Plasma Treatment

Plasma-assisted treatment is among the most effective strategies for creating defects in carbon materials by controlling the gas source, power level, and treatment duration. The high-energy plasma environment interacts dynamically with the carbon structure, knocking out carbon atoms to create vacancies and introducing other forms of defects such as interstitials and topological distortions [154,155]. This method allows for the precise tailoring of the defect type and density to meet specific electrochemical performance requirements.

**Figure 8:**
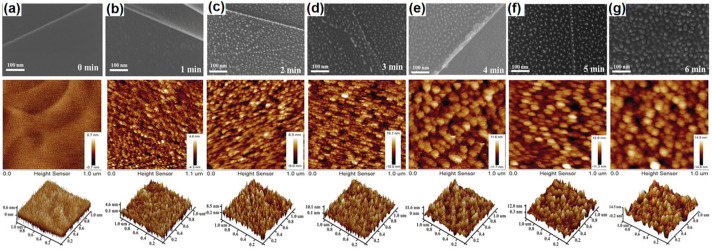
SEM and 2D/3D AFM images of pristine HOPG and plasma-etched HOPG obtained at different times: (**a**) 0 min; (**b**) 1 min; (**c**) 2 min; (**d**) 3 min; (**e**) 4 min; (**f**) 5 min; and (**g**) 6 min [156].

The effectiveness of plasma treatment lies in its ability to control defect densities and types with high precision. Moreover, plasma treatment can modify the surface chemistry of carbon materials without significantly altering their bulk properties, making it a versatile tool for customizing material characteristics for various applications. For instance, Tao et al. utilized argon plasma irradiation to introduce defects into highly oriented pyrolytic graphite (HOPG) [156]. This plasma treatment created numerous defects, such as carbon vacancies and edge sites, significantly increasing surface charge and enhancing electrocatalytic activities for oxygen reduction, oxygen evolution, and hydrogen evolution reactions. As illustrated in the SEM and AFM images (Figure 8a–g), the surface of pristine HOPG was initially smooth. However, after plasma irradiation, the surface progressively became rougher, indicating the formation of rich defects due to the partial removal of carbon atoms. In particular, after 5 min of irradiation, obvious nanocones formed on the surface, primarily due to the strong plasma etching effects. This controllable surface modification provides an ideal model with which to study the relationship between structure, property, and activity. Similarly, Sahoo et al. employed oxygen plasma treatment to introduce oxygen functionalities into vertical graphene nanosheets [157]. This treatment transformed inherently hydrophobic graphene surfaces into super-hydrophilic ones by increasing the presence of hydroxyl and carbonyl groups, resulting in a tenfold increase in areal capacitance and significantly enhancing the electrochemical performance of the supercapacitors.

Further demonstrating the versatility of plasma treatments, Dou et al. highlighted the ability of plasma technology to introduce a variety of defect types [158], including vacancies and edge defects, into different carbon structures, such as graphene and carbon nanotubes. This process not only enhances the specific surface area but also improves the material’s electronic properties by facilitating faster ion transport and better charge storage capabilities. Plasma treatments, such as radio frequency (RF) and dielectric barrier discharge (DBD), are particularly effective for creating these defects without compromising the structural integrity of the materials. The introduction of heteroatoms via plasma treatment can also create active sites that further enhance electrochemical performance.

However, plasma-assisted treatment methods are also expensive, presenting a significant challenge that can hinder their widespread use in industrial-scale applications. Despite this, the unique ability of plasma treatments to introduce a wide range of defects and functional groups makes it a valuable tool for developing advanced carbon-based materials for supercapacitors and other energy storage devices. Future research should focus on optimizing these processes to reduce costs and improve scalability while maintaining the high level of control and precision offered by plasma treatments.

#### 4.1.2. Nitrogen Removal Strategy

The nitrogen removal strategy is a fascinating method by which to construct intrinsic carbon defects, particularly topological defects. This technique employs thermal treatment of nitrogen-doped carbon precursors under inert or controlled atmospheres, facilitating selective nitrogen atom removal. The deliberate elimination of nitrogen atoms results in the creation of vacancies and diverse topological defects within the carbon lattice, such as pentagons, heptagons, and octagons [159]. These defects act as pivotal active sites for charge storage and significantly improve ion transfer kinetics.

One of the principal advantages of denitrogenation lies in its ability to generate a high density of defects without necessitating additional doping or intricate chemical modifications [160]. Furthermore, this method affords meticulous control over the defect concentration through precise adjustments to the annealing temperature and duration. Such control enables the customization of material properties to meet specific requirements of supercapacitor applications. Materials modified via this strategy exhibit enhanced specific capacitance and improved cycle stability, underscoring denitrogenation as a promising pathway for advancing carbon-based supercapacitor technologies.

**Figure 9:**
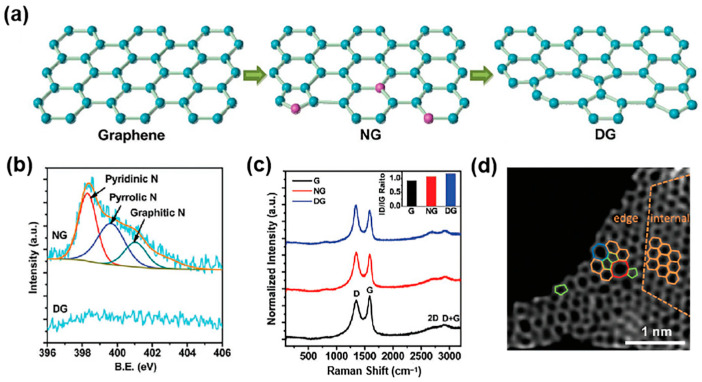
(**a**) The schematic of the formation of DG. (**b**) High resolution of N1s spectra of NG and DG. (**c**) Raman patterns of pristine graphene, NG, and DG. (**d**) High-angle annular dark-field scanning transmission electron microscopy (HAADF) image of DG with an acceleration voltage of 80 kV. Hexagons, pentagons, heptagons, and octagons are labeled in orange, green, blue, and red, respectively [161].

Empirical evidence of the efficacy of denitrogenation includes work by Jia et al., who synthesized defect graphene (DG) using a high-temperature nitrogen removal process from a N-doped precursor [161]. A schematic illustration (Figure 9a) depicts the removal of nitrogen atoms, leading to the formation of defects such as pentagons, heptagons, and octagons due to the reconstruction of the carbon lattice from single-atom vacancies induced after nitrogen removal. These defects arise from the reconfiguration of the carbon lattice to minimize energy following nitrogen removal, potentially altering the local electronic environment of graphene and enhancing its electrochemical reactivity. Additionally, X-ray photoelectron spectroscopy (XPS) analysis confirmed the loss of nitrogen dopant after heat treatment, with a notable reduction from approximately 4 at.% nitrogen in the starting N-doped graphene (NG) to negligible amounts in the defect graphene (DG) (Figure 9b). This change was accompanied by an increase in the intensity ratio of the D band to the G band in the Raman spectra (Figure 9c), indicating a higher defect density post-treatment. Aberration-corrected high-resolution transmission electron microscopy (AC-HRTEM) images (Figure 9d) visualized these defects, showing various combinations of pentagons, heptagons, and octagons near the lattice vacancies, predominantly at the edges of the holes created by nitrogen removal.

Additionally, Dong et al. employed ammonia thermal treatment to remove pyrrolic-N and pyridinic-N dopants from nitrogen-enriched porous carbon effectively [162]. This treatment led to the formation of high-density topological defects, thereby generating crucial active sites within the carbon lattice. These sites play a vital role in optimizing the catalytic performance for carbon dioxide electroreduction, illustrating the broad applicability of denitrogenation in enhancing the functionality of carbon-based materials.

The nitrogen removal strategy also contributes to creating a highly defective yet stable carbon structure. For example, removing nitrogen atoms from the carbon matrix induces vacancies that eventually form more complex defects like G585 structures. These defects enhance the density of states at the Fermi level, significantly improving the material’s quantum capacitance and overall electrochemical performance. Zhao’s group have demonstrated that divacancies and other complex topological defects can enhance oxygen reduction reaction (ORR) activities, providing an effective alternative to nitrogen-doping mechanisms [163]. Their study highlighted that these defects can act as robust active sites for electrochemical reactions, thus enhancing the material’s performance in energy storage applications.

Moreover, nitrogen removal not only generates vacancies but also impacts the electronic properties of the carbon material [164]. By reconfiguring the local electronic environment, these vacancies and defects facilitate improved charge transfer and storage capabilities. High-temperature treatment in an inert atmosphere, as shown by several studies, has been effective in producing stable defect structures that significantly enhance the electrochemical properties of the material. This strategy allows for the precise tailoring of defect densities, enabling the optimization of supercapacitor performance without compromising material stability [161,163].

In summary, the nitrogen removal strategy is a powerful method for creating intrinsic carbon defects, which play a crucial role in enhancing the performance of carbon-based supercapacitors. This approach not only generates a high density of defects but also allows for precise control over defect types and concentrations. As research progresses, further optimization and understanding of this strategy will continue to drive advancements in supercapacitor technology, leading to more efficient and reliable energy storage solutions.

#### 4.1.3. Ball Milling

Ball milling is a highly effective mechanical technique for introducing intrinsic defects into carbon materials, enhancing their electrochemical properties for applications like energy storage and catalysis [165]. This process involves the rotation of a high-energy ball mill at variable speeds, causing vigorous movement of the balls within a grinding jar. As these balls collide with the carbon material, mechanical stress is applied, resulting in the fracturing and deformation of the carbon structure [166,167].

**Figure 10:**
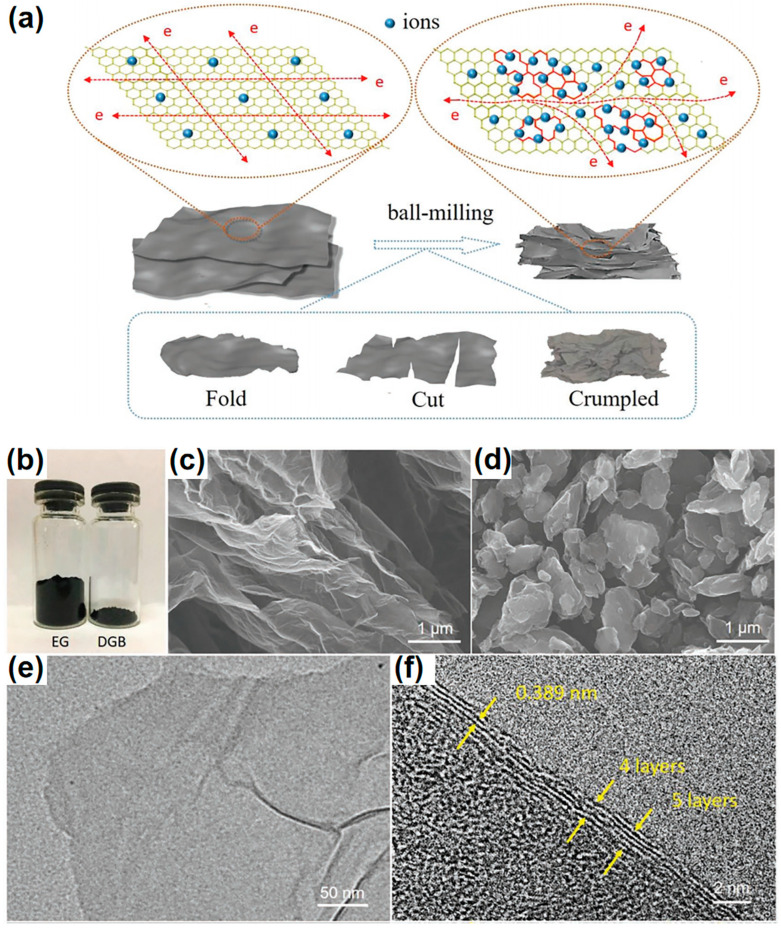
(**a**) The structural evolution of EG during ball milling. (**b**) Photograph of EG and DGB with the same weight of 100 mg. (**c**,**d**) SEM images of EG and DGB. (**e**,**f**) TEM images of EG and DGB [168].

The primary advantage of ball milling is its ability to create a wide variety of defects, including vacancies, dislocations, and grain boundaries. These defects increase the surface area and active sites available for electrochemical reactions, crucial for improving the performance of devices such as batteries and supercapacitors. For example, Dong et al. successfully utilized this method to derive defect-enriched dense graphene blocks (DGBs) from expanded graphene (EG), incorporating extensive “self-doping” defects while maintaining the chemical composition unchanged [168]. The structural transformation of EG to DGB through ball milling is illustrated in Figure 10a. The process involves the flat and highly crystallized EG platelets being folded, cut, and reassembled during ball milling, along with the introduction of abundant “self-doping” defects. This transformation results in the formation of dense DGB with a novel “river basin”-like defect distribution. The “self-doping” defects act as active sites for ion storage with an electrical double-layer behavior, while the highly crystallized regions allow fast electron transport throughout the block. Figure 10b demonstrates the densification from fluffy EG to compact DGB, with the dramatically increased packing density from 0.075 to 0.917 g cm^−3^. SEM images (Figure 10c,d) further show the loose stacked thin-layered EG being crushed into small fragments and reassembled into dense blocks through ball milling, with some mechanical shearing cracks visible at the edge of the DGB flakes (Figure 10e,f).

The process of ball milling not only introduces defects but also transforms the structural and morphological characteristics of the carbon materials. The high-energy mechanical shock during ball milling creates “self-doping” defects, leading to the formation of a unique sp^2^/sp^3^ hybridized structure. This structure ensures abundant active sites for energy storage and maintains good electron transport ability due to fast electron transport in the interconnected sp^2^ crystallized “rivers”. Moreover, the parameters of the ball-milling process, such as the speed, duration, and type of ball used, can be finely tuned to control the extent and type of defects introduced [169]. This level of control makes ball milling a versatile and widely used method in the preparation of advanced carbon-based materials, where specific defect profiles are needed to tailor the material’s electrochemical characteristics for specific applications. For instance, varying the ball-milling duration can modulate the defect density and the sp^2^/sp^3^ hybridized structure, as seen in the increased I_D_/I_G_ ratio in Raman spectra, indicating higher defect densities with longer milling times.

In another study, the same ball-milling process was used to prepare defect-rich carbon materials by mechanically breaking down expanded graphene into smaller platelets. This process enriched the material with defects and optimized the sp^2^ cluster size, creating a structure with large interlayer spacings that facilitate fast sodium storage and improved electrochemical performance [170].

In summary, ball milling is an indispensable technique for creating a variety of intrinsic defects in carbon materials. Its ability to finely control the type and density of defects makes it a powerful tool for optimizing the electrochemical properties of carbon-based materials. By leveraging the advantages of ball milling, researchers can develop advanced materials with tailored characteristics that meet the specific demands of energy storage and other electrochemical applications.

To provide a detailed comparison of the methods used to introduce intrinsic defects in carbon supercapacitors, Table 4 summarizes the key aspects of each strategy, including their advantages, disadvantages, and the capacitance performance achieved under specified current densities. These methods each contribute uniquely to the enhancement of electrochemical properties in carbon materials by introducing and controlling intrinsic defects. The choice of method depends on the specific requirements of the application and the desired properties of the final material. Proper management of intrinsic defects can lead to significant improvements in energy density, power density, and overall efficiency in supercapacitors, making these strategies crucial for the advancement of carbon-based energy storage technologies.

### 4.2. Electrochemical Effects of Intrinsic Carbon Defects

Intrinsic carbon defects, along with structural disorder, play a pivotal role in modulating the electrochemical performance of carbon-based supercapacitors. These defects and the structural disorder directly alter the carbon lattice, introducing irregularities that enhance ion transport and storage capabilities [175,176]. Intrinsic carbon defects, which are inherent imperfections within the carbon lattice, can be broadly categorized into vacancies, edge defects, and topological distortions [177].

Vacancies, areas where carbon atoms are absent from their lattice positions, create sites that can trap charge carriers, thereby increasing the material’s capacitance. This disruption in the electronic structure also enhances conductivity and energy storage. Edge defects, found at the boundaries of the carbon lattice, increase the number of active sites for ion adsorption, thereby improving the material’s reactivity. These edge defects, further categorized into armchair and zigzag edges, possess distinct electronic properties that significantly affect electrochemical performance. Topological defects, such as pentagons, heptagons, and other non-hexagonal rings, introduce significant strain and curvature in the material, altering its electronic properties and enhancing the density of states at the Fermi level. This increase in the density of states boosts the quantum capacitance and overall electrochemical performance.

The integration of structural disorder into the discussion of intrinsic defects is crucial, as recent research published in the journal Science has highlighted the strong correlation between structural disorder in nanoporous carbon electrodes and capacitance. The study demonstrated that more disordered carbons with smaller graphene-like domains exhibit higher capacitances due to the more efficient storage of ions in their nanopores [178]. This discovery underscores the importance of considering both intrinsic defects and structural disorder when optimizing the performance of supercapacitors.

These defects and the structural disorder enhance various aspects of the material’s performance, including capacitance, conductivity, and energy storage capabilities. The following discussion delves into the specific electrochemical effects of intrinsic carbon defects and structural disorder, emphasizing their role in increasing capacitance, improving conductivity, and facilitating faster charge–discharge cycles.

#### 4.2.1. Increased Capacitance

One of the most notable effects of intrinsic carbon defects is the increase in capacitance [179]. Defects such as vacancies and edge sites create a higher density of active sites for ion adsorption, which is crucial for enhancing the material’s capacitance. The disruption of the regular carbon lattice by these defects leads to an increase in surface area and reactivity, providing more locations for ion storage [180].

**Figure 11:**
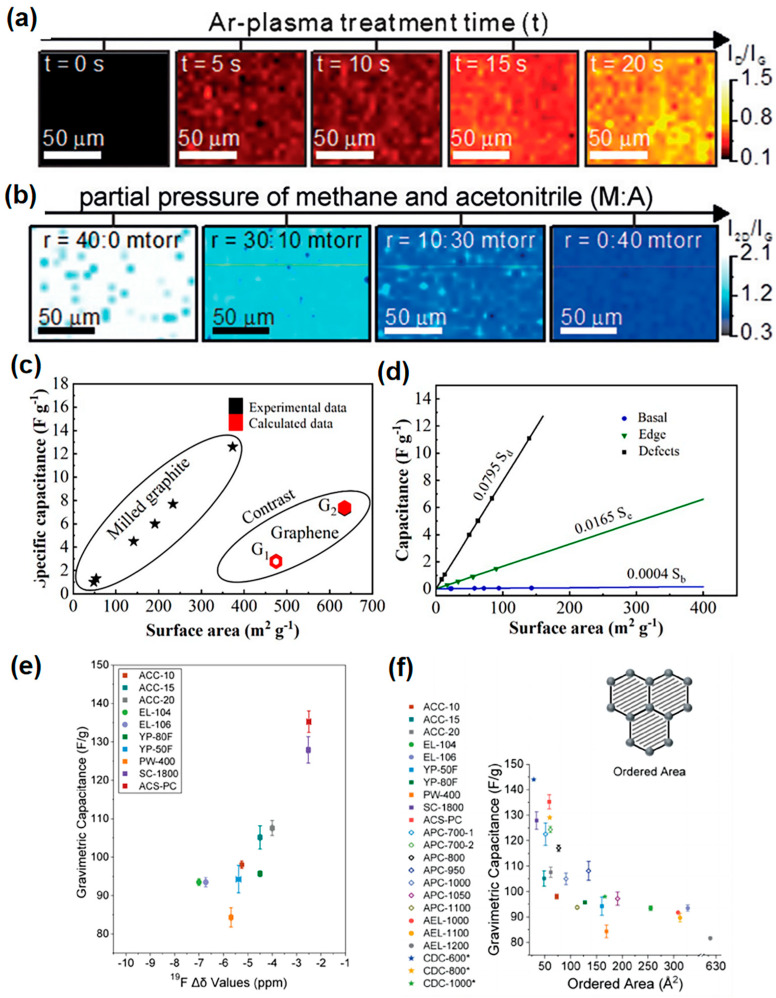
(**a**) Raman maps drawn from the I_D_/I_G_ of the Ar^+^ plasma-treated graphene and (**b**) from the I_2D_/I_G_ of the N-doped graphene [181]. (**c**) Modified non-local density functional theory surface area vs. capacitance (★ represent graphite samples treated with ball milling for different durations; ⬡ and ⬢ represent two types of commercial graphene samples) and (**d**) linear relationship between three types of surface and capacitance [182]. (**e**) Gravimetric capacitance versus ^19^F Δδ values for carbon materials with averaged in-pore shifts. (**f**) Gravimetric capacitance correlates with the calculated size of ordered domains in carbon materials (The asterisks (*) in CDC-600*, CDC-800*, and CDC-1000* indicate that these data points are derived from other research studies and are used for comparison and reference purposes) [178].

Chen et al. have demonstrated that topological defects significantly enhance the quantum capacitance of carbon materials, leading to higher electrochemical double-layer capacitance (EDL) [181]. These defects, such as pentagons and heptagons, introduce significant strain and curvature in the carbon lattice, altering electronic properties and increasing the density of states (DOS) at the Fermi level. This increased DOS facilitates greater charge storage, thereby improving the overall capacitance of the material. The study involved fabricating planar electrodes from CVD-derived single-layer graphene with controlled concentrations of topological defects and nitrogen dopants (Figure 11a,b). The results reveal that, while both topological defects and nitrogen dopants improve the EDL capacitance, they do so through different mechanisms. Topological defects boost quantum capacitance by increasing the DOS, whereas nitrogen dopants enhance capacitance by shifting the Fermi level of graphene. These findings provide insights into the influence of quantum effects on macroscopic properties like EDL capacitance, highlighting the importance of defect engineering in designing advanced supercapacitors with optimized carbon electrodes.

Furthermore, Li’s study demonstrates that the EDLC performance of carbon materials is significantly influenced by different types of wall surfaces [182]. By ball milling graphite powder, basal, edge, and defect surfaces were introduced and quantified using non-local density functional theory. Figure 11c shows the relationship between total wall surface area and capacitance, revealing that, despite G1 and G2 electrodes having higher specific surface areas, their capacitance is lower than that of MG electrodes, indicating a greater contribution from defect surfaces. Figure 11d’s regression analysis shows the unit area capacitance contributions of basal, edge, and defect surfaces to be 0.04 μF cm^−2^, 1.65 μF cm^−2^, and 7.95 μF cm^−2^, respectively. This demonstrates that defect surfaces contribute the most to capacitance, while basal surfaces contribute the least. The study highlights the superior capacitive performance of defect-rich carbon materials for energy storage applications and provides new insights into optimizing carbon electrode charge storage performance.

Building upon these findings, recent research has further elucidated the role of structural disorder in enhancing capacitance. Carbon materials with larger structural disorder, which may correlate with a higher concentration of topological defects, have been found to exhibit higher capacitance. This enhanced performance is attributed to the more efficient storage of ions within the nanopores of these disordered carbons (Figure 11e,f) [178]. The increased charge localization due to smaller structural domains leads to stronger interactions between ions and carbon atoms, thereby improving ion storage efficiency. Consequently, carbons with smaller domains, which are associated with higher concentrations of topological defects such as edge sites, pentagons, heptagons, and curvature, display superior capacitive performance. These discoveries not only reinforce the significance of defect engineering but also extend our understanding to include the broader impact of structural disorder on capacitance, offering valuable guidance for the development of high-performance supercapacitor electrodes.

#### 4.2.2. Enhanced Conductivity

Intrinsic defects also play a crucial role in enhancing the electrical conductivity of carbon-based materials. Vacancies and edge defects disrupt the carbon lattice, creating localized states that can facilitate the movement of charge carriers. This disruption can lower the activation energy required for electron transport, thus improving the overall conductivity of the material [183].

For instance, vacancies introduce localized electronic states that can serve as pathways for electron movement, thereby enhancing conductivity. Edge defects, particularly in graphene and carbon nanotubes, can also significantly impact conductivity. The presence of zigzag and armchair edges introduces variations in electronic band structures, which can either increase or decrease the conductive properties of the material. Generally, these edge defects enhance the material’s ability to conduct electrons, contributing to better performance in supercapacitors.

#### 4.2.3. Improved Charge–Discharge Rates

The presence of intrinsic carbon defects facilitates faster charge–discharge cycles in supercapacitors. This is primarily due to the enhanced ion transport mechanisms provided by these defects. For example, vacancies and edge defects increase the accessibility of ions to the active sites, reducing the diffusion distance and time required for ions to reach these sites [184,185].

Studies have demonstrated that materials with a high density of intrinsic defects exhibit significantly improved rate capabilities. This improvement is attributed to the increased number of active sites and the enhanced pathways for ion and electron transport created by the defects. The rapid movement of ions and electrons through these pathways allows for quicker charging and discharging of the supercapacitor, making it more efficient for high-power applications [186].

#### 4.2.4. Stability and Durability

While intrinsic defects enhance the electrochemical properties of carbon materials, they can also influence the stability and durability of supercapacitors. The introduction of defects can sometimes lead to structural weaknesses that may affect the long-term stability of the material. However, by carefully controlling the type and density of defects, it is possible to balance enhanced performance with stability.

For instance, controlled introduction of edge defects and vacancies can enhance performance without significantly compromising the material’s integrity. Research has shown that optimizing the defect density is key to maintaining the structural stability of the electrode material while achieving high capacitance and conductivity [187].

Overall, intrinsic carbon defects play a crucial role in enhancing the electrochemical performance of carbon-based supercapacitors [188]. By increasing the density of active sites, improving conductivity, and facilitating faster charge–discharge cycles, these defects significantly contribute to the overall efficiency and effectiveness of supercapacitors. Advanced formation strategies, such as plasma treatment, acid treatment, thermal methods, and ball milling, provide precise control over defect structures, enabling the development of high-performance energy storage devices.

As research progresses, the optimization and understanding of intrinsic defects will continue to drive innovations in supercapacitor technology, leading to more efficient and reliable energy storage solutions. Future research should focus on developing advanced characterization techniques to better understand the relationship between defect structures and electrochemical performance. Additionally, scalable and environmentally friendly methods for introducing and controlling intrinsic defects should be explored to ensure sustainable production of high-performance supercapacitors. This ongoing exploration will be vital in ensuring the widespread application and environmental compatibility of next-generation supercapacitors.

## 5. Surface and Interface Engineering

In the realm of supercapacitor electrode engineering, enhancing the surface activity of electrodes and their interaction with electrolytes is crucial for improving performance. Surface and interface engineering aim to modify the surface properties of electrode materials to boost their capacitance, conductivity, and stability. This can be achieved through various techniques, such as coating with conductive polymers, applying metal oxides, and introducing chemical functional groups [59]. These modifications not only improve the physical and chemical characteristics of the electrodes but also enhance their compatibility with different electrolytes, thereby significantly enhancing the overall performance of supercapacitors. The subsequent sections will delve into specific methods of electrode surface modification and chemical functionalization, highlighting their impact on supercapacitor performance.

### 5.1. Electrode Surface Modification

Adopting specific materials and techniques to enhance the surface activity of electrodes and their interaction with electrolytes is a key strategy for performance improvement [189]. For instance, using conductive polymers such as polypyrrole or polyaniline as coating materials effectively enhances the conductivity and electrochemical stability of the electrode surfaces [190]. These polymers not only possess excellent conductivity but also offer increased surface area and more active sites due to their unique microstructures, thereby boosting ion adsorption and charge transfer efficiency. For example, Pattananuwat et al. have developed a one-pot hydrothermal method to synthesize a ternary composite of polyaniline wrapped reduced graphene aerogel and silver nanoparticles (PANi/rGA/AgNPs) [191]. This composite leverages the synergistic effects of the large surface area of the reduced graphene aerogel, the high redox activity of silver nanoparticles, and the conductivity of polyaniline to enhance the rate capability and cycle stability of solid-state supercapacitors. The resulting material demonstrates a specific capacitance of 365.14 F g^−1^ at 0.5 A g^−1^ and retains 88% of its capacity after 4000 cycles at 1 A g^−1^, showcasing a significant improvement in the performance of supercapacitor devices. Zaulkiflee et al. demonstrated the potential of PANI to enhance supercapacitor performance by utilizing a one-step electrochemical polymerization method to coat PANI on various substrates, including carbon, stainless steel, and carbon felt. This method significantly improved the processability, conductivity, and stability of PANI for supercapacitor applications. The study revealed that the highest specific capacitance was achieved with PANI-coated carbon felt, which exhibited enhanced electrochemical performance due to the synergistic relationship between PANI’s good porosity and rapid pseudocapacitive charge storage response, and the high conductivity and stability provided by the carbon felt substrate [192].

**Figure 12:**
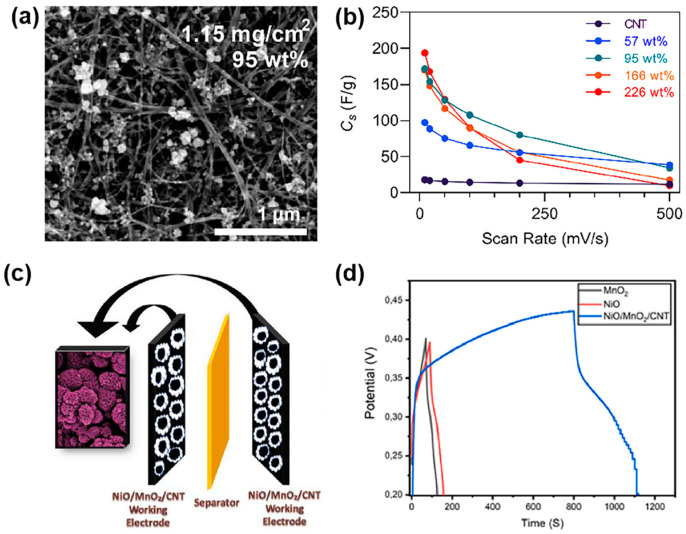
(**a**) SEM top-view images of electroless-treated electrode, (**b**) specific capacitances as a function of CV scan rate for selection of electrode [197]. (**c**) Schematic presentation of the prepared symmetric supercapacitor. (**d**) Galvanostatic charge–discharge (GCD) curves of the electrodes at 1 A g^−1^ current density [198].

Additionally, metal oxides like MnO_2_, NiO and RuO_2_ are commonly employed for electrode surface modification [193,194]. These materials are extensively studied due to their excellent pseudocapacitive properties and chemical stability. For example, coating a thin layer of MnO_2_ on carbon nanotubes or graphene can significantly enhance the specific capacitance of the electrodes. MnO_2_ contributes additional faradaic pseudocapacitive reactions and improves the hydrophilicity of the electrode surface, which facilitates electrolyte penetration and accelerates ion transport [195,196]. In a related study by Tynan et al., MnO_2_ was electrolessly deposited onto carbon nanotube (CNT) mats [197]. This method allowed for a uniform integration of MnO_2_ throughout the porous structure of the CNTs (Figure 12a), improving mechanical strength and stiffness while reducing electrical resistance across the electrode. The optimal loading of MnO_2_ increased the total capacitance of the electrode material by nine times compared with the baseline material (Figure 12b), demonstrating a substantial improvement in device-level performance. Furthermore, In the research conducted by Peçenek et al., the utilization of a composite material comprising MnO_2_, NiO, and carbon nanotubes (CNTs) significantly improved both the physical and electrochemical properties of supercapacitor electrodes [198]. The combination facilitated a unique flower-like morphology, which enhanced the structural integrity and increased the surface area available for electrochemical reactions (Figure 12c). This structural advantage, coupled with the synergistic effects of the metal oxides and carbon materials, led to a high specific capacitance of 1320 F g^−1^ at a current density of 1 A g^−1^ (Figure 12d). Additionally, the supercapacitor demonstrated remarkable cycle life, maintaining over 90% of its initial capacitance after 3000 charge–discharge cycles.

The innovations in electrode surface engineering underscore a transformative impact on supercapacitor technology, highlighting the critical role of surface modifications in achieving superior performance. However, optimizing these electrode materials presents challenges, such as ensuring uniform coating and maintaining structural integrity during repeated charge–discharge cycles. Future research must focus on refining these methods to fully harness their potential, addressing issues like scalability and cost-effectiveness to advance the capabilities and applications of supercapacitors in various high-demand sectors. This ongoing work is crucial for developing next-generation energy storage solutions that meet the growing demands for higher performance and reliability.

### 5.2. Chemical Functionalization

Chemical functionalization of supercapacitor electrode surfaces represents a strategic approach designed to enhance electrochemical performance by improving the interface between electrodes and electrolytes [199]. This method involves the introduction of functional groups that alter the chemical and physical properties of the electrode surface.

**Figure 13:**
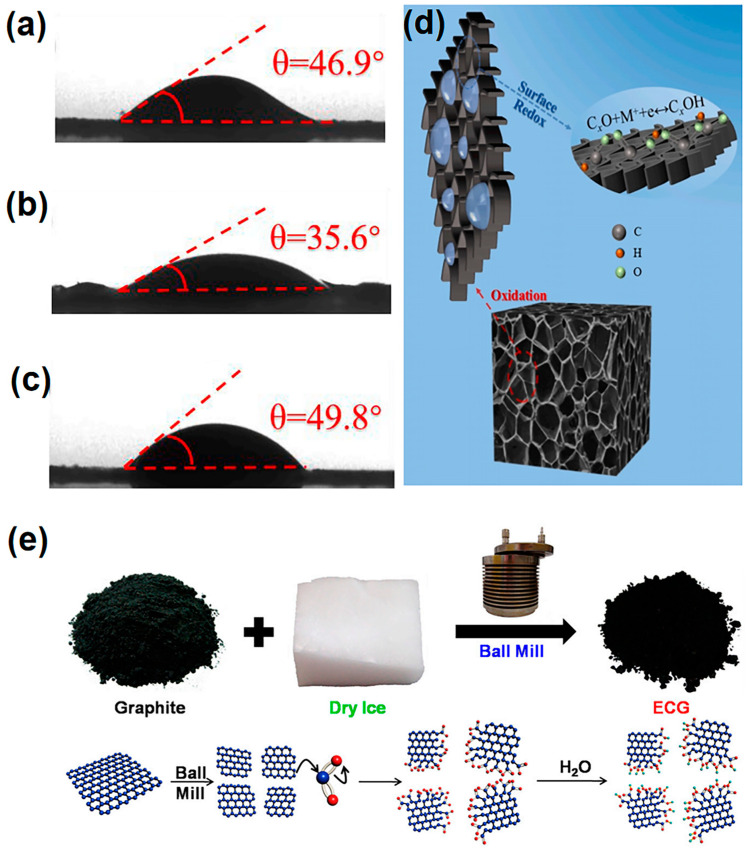
Contact angle test of (**a**) OAC-0.5, (**b**) OAC-1, and (**c**) OAC-1.5 samples. (**d**) The structure diagram of the experimental oxidation process explains the active role of C-O and COOH groups in the surface pseudo capacitance reaction [61]. (**e**) A schematic representation of physical cracking and edge-carboxylation of graphite by ball milling with dry ice, followed by protonation upon exposure to air moisture [201].

Typically, functional groups such as hydroxyl (–OH), carboxyl (–COOH), and amino (–NH_2_) groups are introduced onto the surface of carbon materials through various chemical reactions [60]. These groups enhance the hydrophilicity of the electrodes, significantly improving wettability and ensuring better contact with the electrolyte. Enhanced wettability facilitates the adsorption and desorption of ions on the electrode surface, thereby increasing the capacitance and energy density of supercapacitors [200]. Li and colleagues introduced oxygen-containing functional groups such as C-O and COOH to activated carbon, resulting in the creation of oxidized activated carbon (OAC), through thermal treatment strategies. This modification enhanced the wettability between the electrode material and the electrolyte (Figure 13a–c), significantly improving ion accessibility and pseudocapacitance (Figure 13d). Moreover, the presence of these functional groups facilitated enhanced ion conductivity and more efficient charge transfer processes, leading to a notable increase in the overall electrochemical performance of the supercapacitors [61].

Peng and colleagues extensively reviewed various strategies for attaching functional groups, such as hydroxyl (–OH), amino (–NH_2_), and carboxylic (–COOH) groups, to graphene, employing both covalent and non-covalent methods. These methods enhance graphene’s electrochemical performance by significantly improving its surface polarity and electrolyte accessibility. Covalent approaches, like chemical oxidation and 1,3dipolar cycloaddition reactions, create strong, permanent bonds that alter the electronic properties of graphene, facilitating improved charge storage and transfer. Non-covalent methods, on the other hand, involve physical interactions that modify the graphene surface without altering its structure, which is crucial for maintaining the inherent properties of graphene while enhancing its functionality. These functionalization techniques lead to increased ion transport and interaction with the electrolyte, significantly enhancing the pseudocapacitance and overall energy storage capabilities of supercapacitors [60]. For instance, Jeon et al. conducted significant research on edge-carboxylated graphene nanosheets (ECG) using a ball-milling technique [201]. This method involved ball milling pristine graphite in the presence of dry ice (solid CO_2_) to achieve high-yield edge-selective carboxylation without affecting the basal plane (Figure 13e). The process resulted in the production of highly dispersible ECG in various solvents, which could self-exfoliate into single- and few-layer graphene nanosheets. These ECGs exhibited superior electrical conductivity and could be used for fabricating large-area graphene films, demonstrating their potential for various applications, including supercapacitors. The carboxylation at the edges enhanced the electrochemical properties of the graphene, making it a promising material for energy storage devices.

Overall, chemical functionalization is pivotal for tailoring electrode surfaces to specific electrolytes or operational conditions, optimizing supercapacitors for various applications. This approach leverages the intrinsic properties of electrode materials and aligns with advancements in material science, playing a crucial role in the evolution of high-performance energy storage technologies. Future research should focus on addressing challenges such as the stability of functionalized groups and their long-term electrochemical performance, aiming for sustainable and efficient energy storage solutions.

To further illustrate the practical implications and performance variations resulting from different surface and interface engineering approaches, Table 5 is presented below. This table comprehensively showcases various electrode material modification strategies within the realm of surface and interface engineering, offering a clear overview of the key parameters and details associated with each strategy.

In the enhancement of carbon-based supercapacitor performance, pore structure optimization, heteroatom doping, intrinsic defect engineering, and surface/interface engineering each play distinct yet essential roles. They are interconnected and exert synergistic effects on supercapacitor performance, while also encountering unique challenges.

Pore structure optimization is dedicated to constructing hierarchical pore architectures or modulating pore size distributions. Its primary advantage is the creation of efficient pathways for ion transport, which significantly reduces ion diffusion resistance and thereby markedly improves capacitance performance and power density. However, this strategy has a relatively limited impact on the intrinsic electronic structure of electrode materials, and its role in enhancing the inherent electrochemical performance of materials is not as pronounced. Heteroatom doping primarily modifies the electronic structure of electrode materials to introduce pseudocapacitance, thus increasing energy density. Yet, excessive doping levels of heteroatoms may compromise the stability of the crystal structure, adversely affecting the material’s electrochemical performance. Intrinsic defect engineering enhances electrochemical active sites by introducing defects such as vacancies and edges, promoting ion adsorption and charge transfer. Nevertheless, the introduction of defects can, to some extent, impair the structural integrity of the material, posing a threat to its long-term cycling stability. Surface/interface engineering seeks to strengthen the interaction between electrodes and electrolytes, improving wettability and ion adsorption/desorption efficiency through electrode surface modification (e.g., coating with conductive polymers or metal oxides) and chemical functionalization (introducing functional groups). However, in practical applications, challenges such as ensuring coating uniformity and the stability of functionalized groups may arise.

In summary, these strategies operate in concert to influence carbon-based supercapacitors. Pore structure optimization facilitates ion transport, enhancing the effectiveness of heteroatom doping and intrinsic defect engineering; heteroatom doping and intrinsic defect engineering alter the material’s electronic structure and surface properties, synergizing with surface/interface engineering to further augment the interaction between electrodes and electrolytes. Nonetheless, each strategy has areas for improvement when applied individually. Future research should comprehensively consider these factors, exploring pathways for the synergistic optimization of multiple strategies to continuously elevate the performance of carbon-based supercapacitors.

## 6. Conclusions and Outlook

In this review, we have delved into the critical aspects of structural regulation and performance enhancement of carbon-based supercapacitors, with a focus on electrode material engineering. The innovations in pore structure optimization, heteroatom doping, intrinsic defect engineering, and surface/interface modifications have collectively advanced the field of supercapacitors, significantly improving their electrochemical properties and application potential.

### 6.1. Summary of Key Findings

1. Pore Structure Optimization: The hierarchical arrangement of micropores, mesopores, and macropores plays a vital role in determining the electrochemical behavior of carbon-based supercapacitors. Methods such as chemical and physical activation, along with templating techniques, have been pivotal in tailoring the pore structure to achieve a balance between high energy storage capacity and rapid charge–discharge rates. The precise control over pore formation has enabled the development of materials with superior specific surface areas and optimized ion transport pathways.

2. Heteroatom Doping: The incorporation of heteroatoms like nitrogen, sulfur, phosphorus, and boron into carbon matrices has proven to enhance the electrochemical performance significantly. These elements improve electrical conductivity, surface wettability, and introduce pseudocapacitive effects, thereby boosting the overall capacitance and energy density. The use of post-treatment and in-situ doping strategies has facilitated the fine-tuning of these properties, contributing to the development of high-performance supercapacitors.

3. Intrinsic Defect Engineering: The creation of intrinsic defects, such as vacancies, edge defects, and topological distortions, has emerged as a crucial strategy for enhancing the electrochemical properties of carbon materials. Techniques like plasma treatment, thermal annealing, and ball milling have been effective in introducing and controlling these defects, thereby increasing active sites for ion adsorption and improving charge storage capabilities. The ability to tailor defect densities precisely has led to significant improvements in both energy and power densities.

4. Surface and Interface Modifications: Surface engineering techniques, including the coating of carbon electrodes with conductive polymers and metal oxides, have been instrumental in enhancing the electrochemical performance of supercapacitors. These modifications improve surface properties, facilitate ion transport, and introduce additional pseudocapacitive reactions, thereby enhancing the overall stability and efficiency of supercapacitors.

### 6.2. Challenges and Future Directions

Within the field of carbon-based supercapacitors, despite significant advancements, key challenges remain that necessitate in-depth and specialized research if they are to overcome:

1. Scalability of Advanced Techniques: A significant challenge lies in the scalability of the sophisticated fabrication methods that enhance supercapacitor performance. Techniques such as plasma treatment and precision doping, while effective, are currently limited by high costs and complexity, posing barriers to industrial scalability. Future research endeavors should be directed towards the creation of cost-efficient and scalable methodologies that can produce high-performance carbon-based materials suitable for widespread application.

2. Long-Term Stability of Functionalized Materials: The durability of materials that have undergone surface functionalization or doping is another critical challenge. These materials must retain their enhanced properties throughout extended cycles of operation to be viable for practical use. Thus, future research should prioritize the enhancement of material stability and the development of strategies to ensure long-term cycling stability without performance degradation.

3. Fundamental Understanding of Electrochemical Performance: There is also a pressing need for a more profound comprehension of the underlying mechanisms that dictate the electrochemical behavior of supercapacitor materials. Utilizing advanced characterization techniques, coupled with robust theoretical modeling, will be pivotal in unraveling the intricate relationships between material structure and performance. This knowledge is essential for the strategic design of next-generation supercapacitors that are not only efficient but also sustainable.

### 6.3. Conclusions

The field of carbon-based supercapacitors is poised for significant advancements driven by innovations in material engineering. The strategies discussed in this review offer valuable insights into optimizing the structure and performance of supercapacitor electrodes. As research continues to address the current challenges and explore new avenues, the development of next-generation supercapacitors with higher energy and power densities, improved stability, and cost-effective production methods is within reach. These advancements will play a pivotal role in meeting the growing demands for efficient and sustainable energy storage solutions.

## Figures and Tables

**Table 1:** Carbon-based electrode materials: properties, preparation, performance, and applications.

Electrode Material	Microstructural Characteristics	Preparation Methods	Electrochemical Performance Advantages	Cost and Availability	Application Examples or Research Cases	Ref.
Activated carbon	Abundant pores, high specific surface area.	Physical/chemical activation.	Many ion adsorption sites for double-layer capacitance.	Low cost, widely available, commercialized.	Small electronic device power storage.	[35,36]
Graphene	Two-dimensional single/multi-layer of carbon atoms, high conductivity.	Mechanical exfoliation, SiC decomposition, CVD, electrochemical reduction.	Good conductivity, large area for charge/ion transfer.	High cost, challenging to scale up.	High-performance supercapacitor research.	[41,42]
Carbon nanotube	Tubular (single/multi-walled), good conductivity.	CVD, arc discharge, laser ablation.	Unique structure for ion diffusion and charge transfer.	Complex prep, high cost, specific apps.	High-power density supercapacitors.	[38,39]
Carbon aerogel	Three-dimensional porous network, large surface area, low density.	Sol-gel with drying techniques.	Good for ion diffusion/storage and charge transfer.	Complex and costly, high-end potential.	Aerospace power systems.	[45,46]
Carbon fiber	Fibrous, high strength/modulus, conductive.	Organic fiber carbonization.	High strength and conductivity for stability.	Cost varies, used at the high end.	Wearable/flexible electronics.	[47,48]

**Table 2:** Comparative analysis of pore formation strategies in carbon-based supercapacitors.

Comparison Aspect	Physical Activation	Chemical Activation	Template Method	In-Situ Template Method
Activation principle	Carbon materials are activated by high-temperature gases (e.g., CO_2_, steam), which react with the carbon material to remove part of the carbon and create pores.	Carbon precursor is impregnated with chemical activating agents (e.g., KOH, H_3_PO_4_), followed by carbonization to form pores.	External templates (e.g., ZnO, SiO_2_) are used during carbonization to create pore structures, with the template removed later via dissolution or other methods.	In-situ templates are used during the synthesis, where the template reacts with the carbon source to form pore structures, and is removed post-synthesis.
Pore structure control	Primarily forms micropores, with some mesopores; limited control over pore size distribution.	Provides better control over pore structure, enabling the formation of micropores, mesopores, and macropores.	Allows precise control over pore size and shape, often creating ordered or hierarchical pore structures.	Capable of creating complex hierarchical pore structures with higher surface area and controlled pore distribution.
Impact on supercapacitor performance	Enhances specific capacitance and conductivity, but with limited control over pore size.	Achieves higher specific surface area and better pore structure control, improving capacitance and cycling stability.	Improves electrochemical performance with well-defined pore structures and high surface area, enhancing energy and power density.	Enables precise control over hierarchical pore structures, further enhancing capacitance, rate capability, and stability.
Advantages	Simple to operate, low cost, suitable for large-scale production, applicable to various feedstocks.	Adjustable pore structure, capable of achieving higher surface area and better electrochemical performance, versatile.	Provides precise pore structure control, yielding ordered, porous carbon materials that improve electrochemical performance.	Capable of creating complex hierarchical structures, offering broader pore control, suitable for high-performance energy storage applications.
Disadvantages	Limited control over pore structure, which may lead to lower specific capacitance and energy density.	Process is somewhat more complex, relying on chemical agents, and may pose environmental pollution risks.	Requires template removal, making the process more complex; template material choice and removal can affect product purity.	Synthesis process is more complex, cost is higher, and template removal may result in material loss or contamination.
Example research work	[51,79]	[64,65]	[81,82]	[89,90]

**Table 3:** Impact of heteroatom doping on supercapacitor performance.

Heteroatoms	Doping Content (%)	Specific Surface Area (m^2^ g^−1^)	Capacitance (F g^−1^)	Scan Rate (A g^−1^)	Ref.
N	4.12	2450	325	1	[129]
N	4.5	1012	245	0.5	[130]
N	4.58	397	203	1	[131]
N	4.15	1633	330	0.5	[132]
P	4.1	756	253	1	[133]
P	0.74	1432	966	1	[134]
P	-	1281	292	0.1	[135]
S	25	1952	325	0.125	[136]
B	4	1503	285	1	[137]
B	3.21	315	307	0.5	[138]
O	14.71	936	197	1	[139]
O	16.5	2866	216	0.5	[140]
N,O	2.3/16.34	2027	295	1	[141]
N,O	2.25/14.09	2508	560	0.5	[142]
N,P	3.1/0.62	2245	321	1	[143]
N,S	1.8/1.5	2690	328	1	[144]

**Table 4:** Strategies for introducing intrinsic defects in carbon-based supercapacitors.

Defect Formation Strategy	About the Strategy	Advantages	Disadvantages	Defect Type	Capacitance	Ref.
Plasma treatment	High-energy plasma is used to interact with carbon materials, creating defects by knocking out carbon atoms and introducing interstitials or topological distortions.	Precise control over defect types and density; Eco-friendly process; Can modify surface chemistry without altering bulk properties	Expensive; Complex equipment; Limited scalability for industrial use	Vacancies, edge defects, topological defects	377 F g^−1 ^ (2 mV s^−1^)	[171]
					1.7 mF cm^−2^ (0.1 V s^−1^)	[157]
Nitrogen removal strategy	Thermal treatment of nitrogen-doped carbon materials in inert or controlled atmospheres removes nitrogen atoms, creating vacancies and topological defects in the carbon lattice.	High defect density; Precise control over defect concentration; No need for additional doping	Requires high-temperature treatment; Costly process	Vacancies, topological defects	155 F g^−1^ (1 A g^−1^)	[172]
					182 F g^−1^ (3 A g^−1^)	[173]
Ball milling	Mechanical grinding using high-energy ball mills introduces defects by applying mechanical stress, fracturing and deforming the carbon structure.	Simple and cost-effective; Can create various defects; Increases surface area and active sites for reactions	Process control challenges; Mechanical stress may damage material	Vacancies, edge defects, topological defects	235 F g^−1^ (1 A g^−1^)	[168]
					168 F g^−1^ (1 A g^−1^)	[174]

**Table 5:** Comparison of electrode material modification strategies and their electrochemical performance parameters in supercapacitors.

Electrode Material	Modification Strategy	Specific Capacitance (F g^−1^)	Scan Rate	Energy Density (Wh kg^−1^)	Ref.
PANi/rGA/AgNPs	Coating with conductive polymer polyaniline on reduced graphene aerogel and silver nanoparticles.	365.14	0.5 A g^−1^	116	[191]
PANI-coated carbon felt	One-step electrochemical polymerization method to coat polyaniline on carbon felt.	251.6	20 mV s^−1^	-	[192]
MnO_2_-coated CNTs	Applying a thin layer of MnO_2_ on carbon nanotubes.	192	10 mV s^−1^	0.078	[197]
MnO_2_/NiO/CNTs	Utilizing a composite material of MnO_2_, NiO, and carbon nanotubes.	1320	1 A g^−1^	0.15	[198]
Oxidized activated carbon (OAC)	Introducing oxygen-containing functional groups C-O and COOH through thermal treatment strategies	264	0.5 A g^−1^	-	[61]

## Abbreviations

The following table describes the significance of various abbreviations and acronyms used throughout the thesis.

**Abbreviations**	**Definition**
AC	Activated carbon
ACAs	Activated carbon aerogels
AC-HRTEM	Aberration-corrected high-resolution transmission electron microscopy
AFM	Atomic force microscopy
ACFs	Activated carbon fibers
APC	Activated porous carbon
CAs	Carbon aerogels
CDC	Citric acid-derived carbon
CNS	Carbon nanospheres
CNT	Carbon nanotube
COF	Covalent organic framework
CVD	Chemical vapor deposition
DBD	Dielectric barrier discharge
DES	Deep eutectic solvents
DFT	Density functional theory
DG	Defect graphene
DGB	Defect-enriched dense graphene blocks
DOS	Density of states
ECG	Edge-carboxylated graphene nanosheets
EG	Expanded graphene
GCD	Galvanostatic charge–discharge
GSC	Green stem of cassava
HAADF	High-angle annular dark-field scanning transmission electron microscopy
HCN	Hollow carbon nanorods
HPC	Hierarchical porous carbon
HPCF	Hollow porous carbon fiber
HTC	Hydrothermal carbonization
HRTEM	High-resolution transmission electron microscopy
HOPG	Highly oriented pyrolytic graphite
hNCNC	Hierarchical nitrogen-doped carbon nanocage
OAC	Oxidized activated carbon
ORR	Oxygen reduction reaction
PANI	Polyaniline
PPy	Polypyrrole
PHP	Phosphoric acid plus hydrogen peroxide
PHPLC	PHP lignin-derived carbons
RF	Radio frequency
SEM	Scanning electron microscopy
SSA	Specific surface area
